# The insulin-like peptides Dilp2 and Dilp6 exhibit divergent responses to dietary sugar and protein in *Drosophila* larvae

**DOI:** 10.1073/pnas.2426930122

**Published:** 2025-10-15

**Authors:** Miyuki Suzawa, W. Kyle McPherson, Kelly E. Dunham, Elizabeth E. Van Gorder, Shivani Reddy, Leila A. Jamali, Michelle L. Bland

**Affiliations:** ^a^Department of Pharmacology, University of Virginia, Charlottesville, VA 22908-0875

**Keywords:** insulin signaling, metabolism, growth, dietary nutrients, *Drosophila*

## Abstract

Nutrient intake drives secretion of insulin and insulin-like peptides that stimulate anabolic metabolism and tissue growth. Eight *Drosophila* insulin-like peptides (Dilps) are encoded in the *Drosophila* genome; whether these Dilps respond uniformly to changes in dietary nutrients is unknown. Here, we investigate the endocrine responses of Dilp2, secreted by brain insulin-producing cells, and Dilp6, produced by the fat body, to dietary sugar and protein in mid-third instar *Drosophila* larvae. Starvation leads to a profound reduction in circulating Dilp2 without affecting circulating Dilp6 levels. Diets containing sugar alone drive nutrient storage and increase hemolymph Dilp6, but do not promote Dilp2 release. In contrast, dietary protein drives growth and restores hemolymph Dilp2 but strongly reduces circulating Dilp6. Furthermore, circulating levels of Dilp2 and Dilp6 are modulated by the ratio of sugar to protein in the diet. We find that depleting circulating Dilp6 via fat body specific knockdown or increasing insulin receptor (InR) levels in fat body leads to increased levels of triglyceride storage but decreased peripheral growth. Our results suggest that Dilp6, a hormone produced in response to dietary sugar, may direct the use of sugar for growth instead of fat storage at the end stage of larval development. Our findings reveal different modes of regulation for Dilp2 and Dilp6 and raise the question of how the single known *Drosophila* InR integrates divergent signals from distinct Dilps to control growth and metabolism.

## Significance

The *Drosophila melanogaster* genome encodes multiple insulin-like peptides (Dilps) that bind to a single-known insulin receptor to control growth and metabolism. Here, we show that dietary nutrients regulate two of these hormones, Dilp2 and Dilp6, in opposite ways. Dietary sugar induces secretion of fat body-derived Dilp6 while dietary protein stimulates secretion of brain-derived Dilp2 but suppresses Dilp6. The existence of multiple Dilps that are regulated in divergent ways by distinct stimuli may provide flexibility in the insulin signaling pathway, allowing *Drosophila* to adapt growth and metabolic processes to prevailing conditions and promoting survival.

Nutrient intake is essential for growth and development, and, in animals ranging from planaria to humans, insulin and insulin-like hormones direct the use of dietary nutrients for macromolecule synthesis, nutrient storage, and cell proliferation (1). In the absence of insulin signaling, animals exhibit profound growth and metabolic defects, highlighting the critical roles of insulin and related hormones in communicating nutrient status between organs and in allocating nutrients to different anabolic pathways.

The larval stage of the *Drosophila* life cycle is characterized by rapid growth and high levels of nutrient storage that prepare the animal for metamorphosis and adult life (2). Eight *Drosophila* insulin-like peptides (Dilps) are encoded in the *Drosophila melanogaster* genome. Dilp1-Dilp6 are thought to drive growth and nutrient storage by binding to the single known insulin receptor (InR) in target organs and activating the canonical phosphatidylinositol-3 kinase-Akt signaling pathway (3–8). The hormones Dilp7 and Dilp8 share homology with relaxins and are thought to bind to the G protein–coupled receptors Lgr3 and Lgr4 to regulate development, secretion of other Dilps, and larval behaviors (9–11). Dilps are expressed in various tissues and at different levels across development. Insulin-producing cells (IPCs) in the fly brain synthesize and release Dilps 2, 3, and 5 into hemolymph as well as at synapses in the central brain (12, 13), while Dilp6 is produced and secreted by the fat body, a liver- and adipose-like organ that also coordinates the humoral response to infection (14–16). During the larval third instar, *Dilp2* transcript levels decline, while *Dilp6* is markedly induced by high levels of ecdysone that precede and trigger metamorphosis (14, 17).

In addition to their unique tissue expression patterns and developmental profiles, Dilp2 and Dilp6 exhibit opposite responses to environmental stressors such as starvation and immune activation. In starved larvae, Dilp2 and Dilp5 are retained in IPCs (18–24), leading to decreased circulating levels of these hormones. In contrast, *Dilp6* transcript levels in larval fat body are induced by starvation on 1% sucrose, suggestive of increased circulating Dilp6 in starved larvae (16). Activation of the Toll innate immune signaling pathway in the larval fat body impairs growth, at least in part, by strongly suppressing hemolymph Dilp6 levels, without altering circulating Dilp2 (15).

Whether Dilp2 and Dilp6 are differentially regulated by specific dietary nutrients is unknown. Here, we measure circulating levels of Dilp2 and Dilp6 and show that hemolymph Dilp2 is markedly reduced by starvation, while Dilp6 levels are maintained. We find that Dilp2 and Dilp6 exhibit opposite responses to dietary sugar and protein. Sugar-only diets suppress Dilp2 but lead to elevated Dilp6 in circulation. However, dietary protein promotes secretion of Dilp2 but strongly reduces hemolymph Dilp6. These endocrine changes occur in parallel to nutrient storage and whole-animal growth that are driven by dietary sugar and protein, respectively. Importantly, our findings also reveal that Dilp2 and Dilp6 are not only regulated by sugar and protein individually, but that each hormone also responds to the ratio of these components in the diet. We find modest effects of Dilp2 in regulating larval nutrient storage and growth. However, our data show that, in the late third instar, Dilp6 antagonizes triglyceride accumulation in the larval fat body while promoting growth, suggesting that Dilp6 may direct the use of dietary sugar for growth processes instead of nutrient storage as animals prepare to undergo metamorphosis.

## Results

**Fig. 1.**
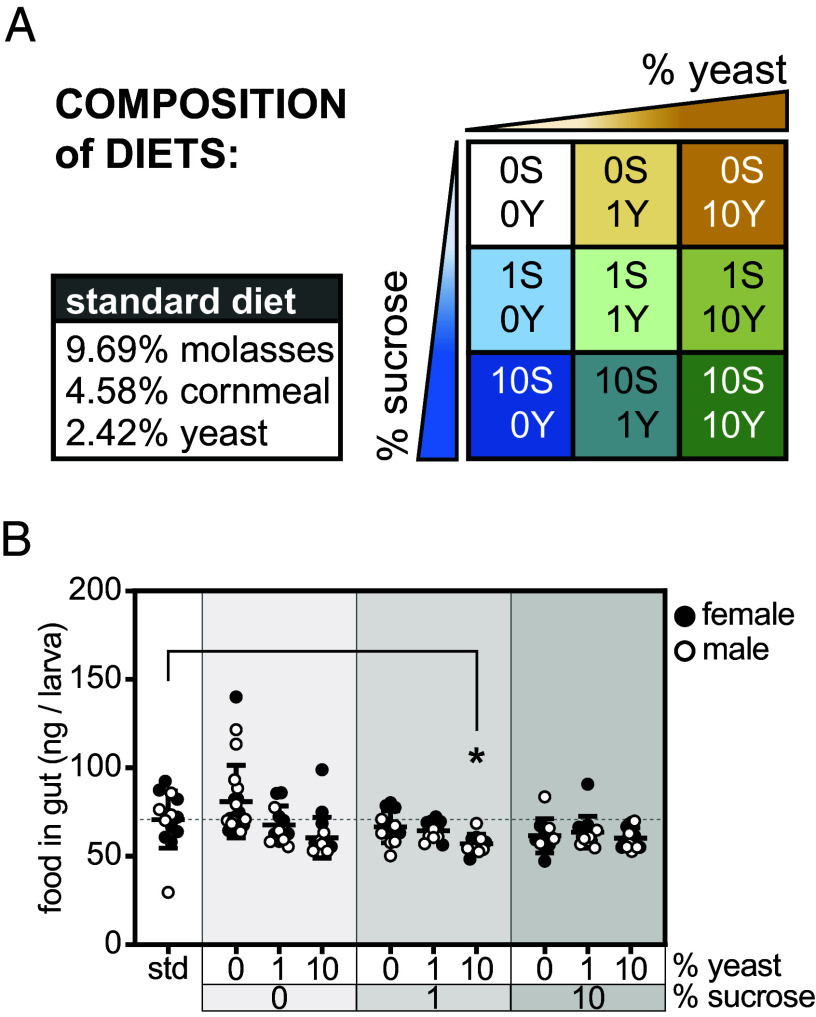
Nutritional geometry approach. (*A*) Composition of standard laboratory diet and nine experimental diets varying dietary sucrose and yeast extract content. (*B*) Quantity of food in gut in *Sgs3-GFP* larvae fed standard and experimental diets containing 1% FD&C Blue No.1 for 24 h, from ~84 to 108 h AEL, n = 4 to 11 samples/sex/diet, with 10 to 21 samples total/diet. Gray shading in the graph indicates sucrose dose; female and male samples are indicated by filled and open symbols, respectively. Data are presented as means ± SD; *P = 0.0484 versus standard diet. *P* values were determined by one-way ANOVA with Dunnett’s multiple comparison test.

To determine how dietary sugar and protein regulate circulating Dilp levels, nutrient storage, and growth, we used a nutritional geometry approach (25, 26). In the wild and in the laboratory, the major components of the *D. melanogaster* diet are fruit sugars and yeast, with the latter serving as the main protein source and supplying cholesterol and lipids (27). Our standard laboratory diet contains 10.97% sugar (from molasses and cornmeal), 1.6% protein (from cornmeal and yeast), and 0.12% fat (from yeast). We prepared nine experimental diets containing sucrose and/or yeast extract at 0%, 1%, or 10% in 0.8% agar (denoted as 0S, 1S, 10S and 0Y, 1Y, 10Y), with yeast extract providing 3% carbohydrate and 63.5% protein by weight (28–30) (Fig. 1*A* and *SI Appendix*, Fig. S1 *A* and *B*).

We examined endocrine, metabolic, and growth responses to dietary nutrients in larvae fed standard and experimental diets during the second half of the third instar, a time of rapid growth and considerable nutrient storage preceding metamorphosis. We staged third instar larvae using Sgs3-GFP, a fusion of the Salivary gland secretion 3 (Sgs3) and green fluorescent protein (GFP) coding sequences expressed under control of the *Sgs3* promoter. Sgs3-GFP expression is induced by ecdysone and first detectable in salivary glands midway through the third instar at ~100 h after egg lay (h AEL). Changes in the pattern and degree of GFP expression allow staging of larvae through the end of the third instar at ~120 h AEL (17, 31). Larvae carrying *Sgs3-GFP* were fed standard and experimental diets beginning in the first half of the third instar stage. After 24 h, Sgs3-GFP expression was scored, and larvae with expression indicating a developmental stage equivalent to ~108 h AEL, a point midway through the second half of the third instar, were selected for further measurements. This ensured that, while a group of third instar larvae with varying developmental time entered the experiment, the animals used for measurements after 24 h on each diet had reached the same developmental stage. Additionally, this approach ensured that larvae used for measurements were subject to dietary manipulations beginning at ~84 h AEL, after attaining critical weight, a point at which *Drosophila* larvae will undergo metamorphosis regardless of the quantity or quality of nutrient intake in the remaining growth period (32–34).

We first asked whether food consumption differed among these ten diets, using diets containing blue food dye, and quantitation of blue food in the gut using spectroscopy of whole-larval lysates. In ~108 h AEL larvae fed diets for 24 h, the quantity of food in the gut at the end of this period did not differ significantly between the standard and experimental diets, except for the 1% sucrose, 10% yeast (1S/10Y) diet (Fig. 1*B*). We observed differences in food intake on experimental diets compared with the standard diet when larvae were transferred to dyed food for 4 h at the beginning, middle, and end stages of the 84 to 108 h AEL feeding period (*SI Appendix*, Fig. S1*C*). At the end of the feeding period (~104 to 108 h AEL), larvae consuming experimental diets exhibited a 26% reduction in gut food content on average compared with larvae reared on standard food (*SI Appendix*, Fig. S1*D*). Our data also suggest that third-instar larvae consume enough food to fill their gut lumens within 4 to 6 h, which is corroborated by the nearly complete transit of blue-dyed food through the gut within 6 h of larvae being transferred to plain agar (*SI Appendix*, Fig. S1 *E* and *F*).

We calculated the energetic content of the food in the gut using standard values of 4 kcal (16.736 kJ) per gram of sugar or protein (*SI Appendix*, Fig. S1*B*). The nine experimental diets provided a range of conditions where calories derived from carbohydrate or protein predominated (*SI Appendix*, Fig. S1*G*). Although differences in gut food content exist among the diets, they are relatively small (81 to 114% of standard diet in larvae fed diets for 24 h) compared with differences in energy derived from carbohydrates (0 to 80% of standard diet) and protein (0 to 340% of standard diet). Therefore, differences in endocrine, metabolic, and growth responses to these diets likely reflect effects of distinct nutrients—carbohydrates and protein—and not food intake per se.

**Fig. 2.**
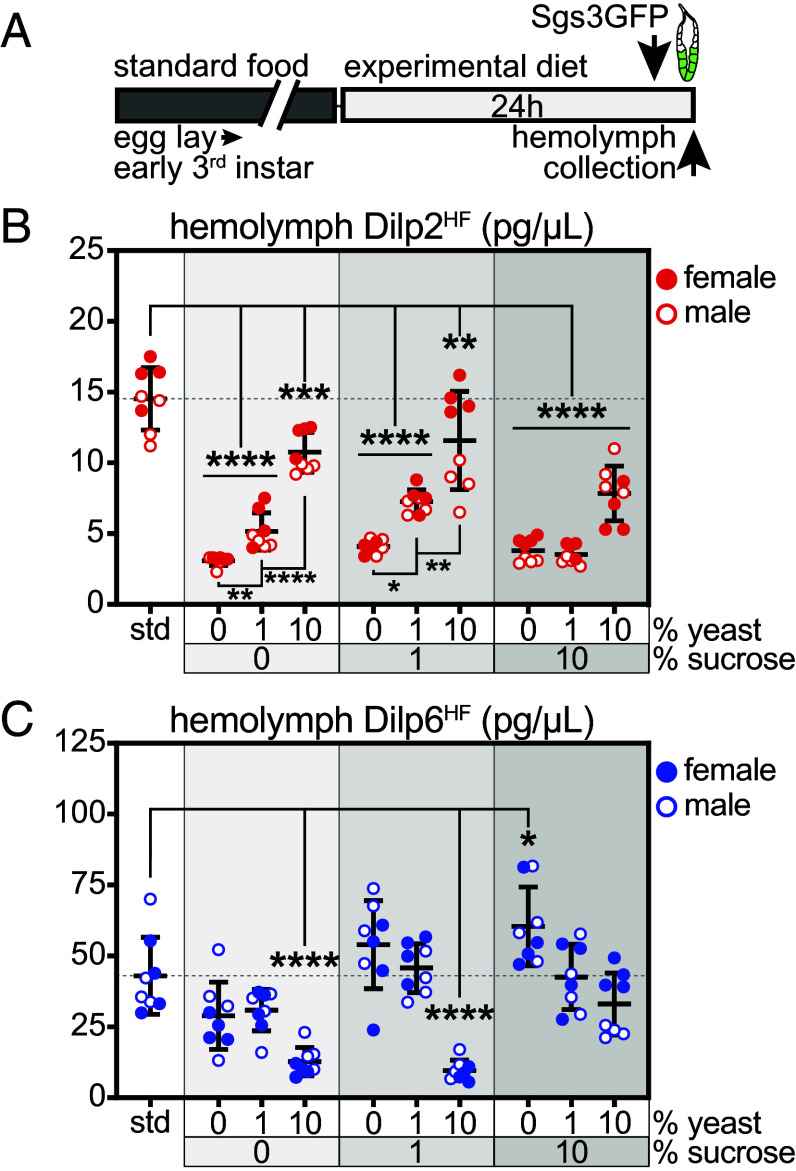
Dilp2 and Dilp6 exhibit divergent responses to dietary sugar and protein. (*A*) Early- to mid-third instar larvae were fed standard food or experimental diets for 24 h, and hemolymph was collected from those with Sgs3-GFP fluorescence indicating progression to ~108 h AEL. (*B*) Hemolymph Dilp2^HF^ in *Dilp2^1^, gDilp2^HF^, Sgs3-GFP* larvae. (*C*) Hemolymph Dilp6^HF^ in *Dilp6^HF^; Sgs3-GFP* larvae. n = 4 samples/sex/group, with eight total samples/group. Gray shading in graphs indicates sucrose dose; female and male samples are indicated by filled and open symbols, respectively. Data are presented as means ± SD; *P ≤ 0.0164, **P ≤ 0.0042, ***P = 0.001, ****P < 0.0001 versus standard diet. *P* values were determined by one-way ANOVA with Dunnett’s multiple comparison test.

To assess the endocrine response to dietary sugar and protein, we used alleles of *Dilp2* and *Dilp6* with HA and FLAG epitope tags inserted into exons encoding the B- and A-chains of each peptide (15, 35). We fed *Dilp2^HF^* and *Dilp6^HF^* larvae standard food or experimental diets for 24 h from ~84 to 108 h AEL, collected hemolymph from ~108 h AEL larvae (staged using Sgs3-GFP), and performed enzyme-linked immunosorbent assays (ELISAs) on pooled hemolymph samples to detect circulating Dilp2^HF^ and Dilp6^HF^ (Fig. 2*A*). Larval brain IPCs retain stored Dilp2 and Dilp5 when larvae are starved and release these Dilps in response to dietary protein (18–24). In agreement with these previous reports, we found that hemolymph Dilp2^HF^ levels were reduced by 79% in response to starvation on plain agar (0S/0Y). Addition of yeast extract (0S/1Y and 0S/10Y) substantially and dose-dependently increased circulating Dilp2^HF^ compared with animals fed plain agar. In larvae fed diets with 1% or 10% sucrose but no added yeast (1S/0Y and 10S/0Y), circulating Dilp2^HF^ levels remained low and indistinguishable from the starved condition. At the highest amount of yeast extract tested, hemolymph Dilp2^HF^ was reduced by 32% when dietary sucrose was increased from 1 to 10% (compare 1S/10Y and 10S/10Y, *P* = 0.0190, Student’s unpaired *t* test). Similarly, we observed a 52% decrease in circulating Dilp2^HF^ levels in larvae fed 1% yeast when sucrose concentrations were increased from 1 to 10% (compare 1S/1Y and 1S/10Y, *P* = 0.0042, Student’s unpaired *t* test) (Fig. 2*B*). Dilp2^HF^ levels responded similarly to dietary nutrients in male and female larvae, but females exhibited higher circulating Dilp2^HF^ levels than males when fed standard food and diets containing 10% yeast with 0% or 1% sucrose (0S/10Y, 1S/10Y) (*P* ≤ 0.05, Student’s unpaired *t* tests). IPCs in the larval brain synthesize not only Dilp2 but also Dilp3 and Dilp5 (12). Transcript levels of *Dilp2* and *Dilp5* in the brain closely paralleled each other and positively correlated with circulating Dilp2 levels (*SI Appendix*, Fig. S2 *A* and *B*). Transcript levels of *Dilp3* in the brain were not strongly modulated by diet (*SI Appendix*, Fig. S2*C*). We found that the presence of the epitope tagged *Dilp2^HF^* or Dilp6*^HF^* alleles led to similar diet-dependent changes in *Dilp2*, *Dilp3*, *Dilp5*, or *Dilp6* transcript levels compared with larvae carrying *Sgs3-GFP* alone (*SI Appendix*, Fig. S2 *E* and *F*).

Dietary nutrients exerted divergent effects on Dilp6 compared with Dilp2. Larvae fed plain agar (0S/0Y) exhibited a 33% reduction in circulating Dilp6^HF^ compared with controls fed the standard diet, although this difference did not achieve statistical significance (*P* = 0.0728). Dietary sucrose led to a 1.25-fold increase (1S/0Y) and a statistically significant 1.4-fold (10S/0Y) increase in circulating Dilp6^HF^ levels relative to larvae consuming standard food. In contrast, larvae fed a diet containing 10% yeast extract in plain agar (0S/10Y) exhibited a 70% decrease in hemolymph Dilp6^HF^ compared with animals fed the standard diet. Moreover, addition of yeast extract to diets containing sucrose led to decreases in hemolymph Dilp6^HF^ such that circulating levels were reduced by 78% (1S/10Y) and 23% (10S/10Y) relative to larvae fed the standard diet. Accordingly, increasing dietary sugar from 1 to 10% in larvae consuming 10% yeast extract led to a 3.4-fold increase in circulating Dilp6^HF^ (compare 1S/10Y and 10S/10Y, *P* < 0.0001, Student’s unpaired *t* test) (Fig. 2*C*). Dilp6^HF^ levels responded similarly to dietary nutrients in male and female larvae, but females exhibited higher circulating Dilp6^HF^ levels than males in the 10S/10Y condition (*P* = 0.0002, Student’s unpaired *t* test). We examined *Dilp6* transcripts levels in fat body, and found that diets containing sucrose alone increased *Dilp6* messenger RNA (mRNA) (1S/0Y and 10S/0Y), as previously reported (16). For most conditions, transcript and circulating levels of Dilp6^HF^ positively correlated (*SI Appendix*, Fig. S2*D*); however, we noted that complete starvation (0S/0Y) led to increased fat body *Dilp6* transcripts but decreased hemolymph Dilp6^HF^ compared with standard food. In summary, our data show that in *Drosophila* larvae, Dilp2 is suppressed by starvation and restored by dietary protein but not sugar. Dilp6 is mildly affected by starvation, decreased in response to dietary protein, and induced by dietary sugar. Additionally, our data suggest that the ratio of dietary protein to sugar is essential for fine-tuning circulating levels of each hormone.

Dilp2 and Dilp6 are regulated by factors including growth-blocking peptides (Gbp) and stunted (sun), both secreted by the fat body (19, 22), and ecdysone, secreted by the prothoracic gland (36). We assessed transcript levels of *Gbp2* and *sun* in fat body in ~108 h AEL larvae fed standard food and experimental diets for 24 h. *Gbp2* expression was not strongly regulated by specific nutrients (*SI Appendix*, Fig. S3*A*). In contrast, *sun* mRNA levels were strongly induced by dietary yeast and closely paralleled circulating Dilp2^HF^ (*SI Appendix*, Fig. S3*B*). As expected, circulating levels of ecdysteroids increased as larvae progressed through the third instar (*SI Appendix*, Fig. S3*C*). We measured hemolymph ecdysteroid levels in larvae (at ~108 h AEL) that had been fed standard food, plain agar (0S/0Y), high sucrose (10S/0Y), high yeast (0S/10Y), or high sucrose and yeast (10S/10Y) for 24 h. Although ecdysteroid levels were approximately 50% lower in larvae fed standard food, hemolymph ecdysteroids did not differ among experimental diets (*SI Appendix*, Fig. S3*D*). These results suggest that differences in circulating Dilp2^HF^ and Dilp6^HF^ between larvae fed high sucrose (10S/0Y) and high yeast (0S/10Y) are not due to accompanying changes in circulating ecdysteroids. Similarly, transcript levels of two ecdysone receptor target genes, *Eip74EF* in fat body and *ImpE1* in wing disc, were not strongly affected by different diets (*SI Appendix*, Fig. S3 *E* and *F*).

In adult flies, fat body-derived Dilp6 suppresses secretion of Dilp2 from IPCs (37), and loss of Dilps 2, 3, and 5 leads to increased *Dilp6* expression (5). In larvae, glial-derived Dilp6 promotes *Dilp5* expression in IPCs (38). To determine whether regulatory interactions between Dilp2 and Dilp6 occur in larvae, we assessed hemolymph Dilp6^HF^ and Dilp2^HF^ in larvae with loss of Dilp2 and Dilp6, respectively. We observed a 1.9-fold increase in circulating Dilp6^HF^ in *Dilp2^1^* null larvae compared with *Dilp2^1^*/+ heterozygotes. Increased hemolymph Dilp6^HF^ was also observed in *Dilp2^1^*/*Dilp2-3*, *Dilp5^3^* mutants, although Dilp6^HF^ levels were variable on this background (*SI Appendix*, Fig. S3*G*). In *Dilp6^68^* null larvae, levels of Dilp2^HF^ were reduced by 72% compared with wild type (*SI Appendix*, Fig. S3*H*). Dilp6 is expressed both in fat body and in brain glia (39, 40), but the fat body is the source of circulating Dilp6 (15). We found that loss of hemolymph Dilp6 via fat body-specific Dilp6 knockdown led to a mild, 27% decrease in circulating Dilp2^HF^ (*SI Appendix*, Fig. S3*I*), suggesting that the positive regulation of Dilp2 by Dilp6 stems mainly from local interactions in the brain. These data also reveal a difference in Dilp2 and Dilp6 interactions between the larval and adult stages.

**Fig. 3.**
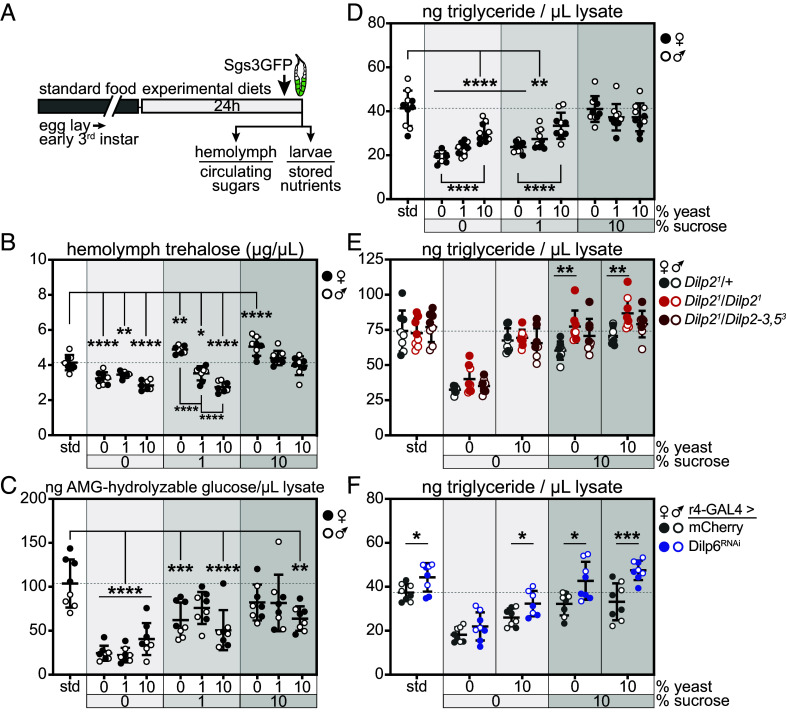
Dietary sugar drives nutrient storage. (*A*) Early- to mid-third instar larvae were fed standard food or experimental diets for 24 h, and those with Sgs3-GFP fluorescence indicating progression to ~108 h AEL were selected for further analysis. (*B*) Hemolymph trehalose levels, n = 4 samples/sex/group, with eight total samples/group. (*C*) Whole-animal glycogen levels, normalized to lysate volume, n = 4 larvae/sex/group, with eight total larvae/group. (*D*) Whole-animal triglyceride levels, normalized to lysate volume, n = 5 larvae/sex/group, with 10 total larvae/group. For panels *B*–*D*, measurements were made in *Sgs3-GFP* larvae. *P ≤ 0.0161, **P ≤ 0.0066, ***P ≤ 0.0009, ****P < 0.0001 versus standard diet. (*E*) Whole-animal triglyceride levels, normalized to lysate volume, n = 3 to 4 larvae/sex/group, with 7 to 8 total larvae/group. All larvae carried *Sgs3-GFP*. **P ≤ 0.0063 versus *dilp2^1^*/+. (*F*) Whole-animal triglyceride levels, normalized to lysate volume, n = 3 to 4 larvae/sex/group, with 7 to 8 total larvae/group. All larvae carried *Sgs3-GFP*. *P ≤ 0.0280 and ***P = 0.0008 versus r4>mCherry. Gray shading in graphs indicates sucrose dose; female and male samples are indicated by filled and open symbols, respectively. Data are presented as means ± SD; *P* values were determined by one-way ANOVA with Dunnett’s multiple comparison test and Student’s unpaired *t* tests.

Insulin signaling regulates carbohydrate, lipid, and protein metabolism in *Drosophila* and throughout the animal kingdom (13, 41–43). We assessed circulating sugars and stored nutrients in larvae raised on standard food or experimental diets for 24 h during the mid-third instar and used Sgs3-GFP to select animals that had reached ~108 h AEL for measurements (Fig. 3*A*). Hemolymph levels of trehalose, the major circulating sugar in *Drosophila*, were highly responsive to dietary conditions (Fig. 3*B*). Larvae fed diets lacking sucrose (0S/0Y, 0S/1Y, 0S/10Y) exhibited 17 to 32% decreases in circulating trehalose compared with animals fed standard food. In contrast, experimental diets containing only sucrose (1S/0Y, 10S/0Y) led to 18 to 22% increases in circulating trehalose. Introducing yeast extract to diets with 1% sucrose reduced hemolymph trehalose in a dose-dependent manner, similar to the pattern observed with hemolymph Dilp6. Glucose circulates at 10-fold lower levels than trehalose. We observed no changes in circulating glucose except for a small increase in larvae fed the 0S/1Y diet (*SI Appendix*, Fig. S4*A*).

Dietary sugar is stored as glycogen in the fat body and body wall muscles and as triglyceride in the fat body. Like circulating trehalose, glycogen storage was driven by dietary sugar. Lysates of larvae fed diets lacking sucrose (0S/0Y, 0S/1Y, 0S/10Y) exhibited 61 to 78% reductions in glycogen compared with those from larvae fed the standard diet. Diets with 1% and 10% sucrose and any quantity of yeast extract partially rescued glycogen storage compared with sucrose-free diets. We also observed a suppressive effect of high levels of dietary protein on glycogen storage in larvae fed 1% or 10% sucrose (1S/10Y and 10S/10Y) compared with animals fed the standard diet (Fig. 3*C*). Whole-animal protein levels were decreased in animals fed 0% or 1% yeast and thereby altered calculation of glycogen stores when glycogen was normalized to protein (*SI Appendix*, Fig. S4*B*). Dietary sugar also drove lipid storage. Lysates of whole larvae fed diets lacking sucrose (0S/0Y, 0S/1Y, 0S/10Y) exhibited 27 to 53% reductions in triglyceride compared with controls fed the standard diet. Addition of sucrose to experimental diets with any quantity of yeast led to increased triglyceride storage. The addition of yeast extract to diets with 0% or 1% sucrose increased triglyceride levels (compare 0S/0Y and 0S/10Y; 1S/0Y and 1S/10Y) but had no effect on triglyceride storage in animals fed diets containing 10% sucrose (Fig. 3*D*). We again observed that whole-animal protein levels were highly responsive to dietary yeast and led to altered calculations of triglycerides when normalized to protein (*SI Appendix*, Fig. S4*C*). The presence of the epitope tagged *Dilp2^HF^* or *Dilp6^HF^* alleles led to similar diet-dependent changes in triglyceride storage compared with larvae carrying *Sgs3-GFP* alone (*SI Appendix*, Fig. S4*D*).

We assessed the roles of brain derived-Dilp2 and fat body-derived Dilp6 in coordinating nutrient storage in the larval stage. We measured triglyceride levels in ~108 h AEL larvae with loss of Dilp2 or Dilp6. These animals were fed the standard diet, or diets lacking sucrose and yeast extract (0S/0Y), with 10% of either ingredient (10S/0Y, 0S/10Y), or 10% of both (10S/10Y) for 24 h beginning in the mid-third instar. In larvae homozygous for the *Dilp2^1^* null allele, triglyceride levels were increased on diets containing 10% sucrose compared with *Dilp2^1^* heterozygotes. Interestingly, triglyceride levels on high sucrose diets were normalized by loss of one copy each of *Dilp3* and *Dilp5* in *Dilp2* null animals (Fig. 3*E*). We used r4-GAL4 to drive fat body-specific expression of a short hairpin RNA targeting Dilp6; this strategy leads to undetectable levels of Dilp6 in hemolymph (15). Larvae with fat body-specific knockdown of Dilp6 exhibited significant increases in triglyceride storage on all diets except plain agar, with 18, 24, 32, and 43% increases in lipid stores on the standard, 0S/10Y, 10S/0Y, and 10S/10Y diets, respectively, compared with control larvae expressing mCherry in fat body (Fig. 3*F*). These results parallel findings of increased lipid storage in adult female *Dilp6^41^* null mutants (5).

**Fig. 4.**
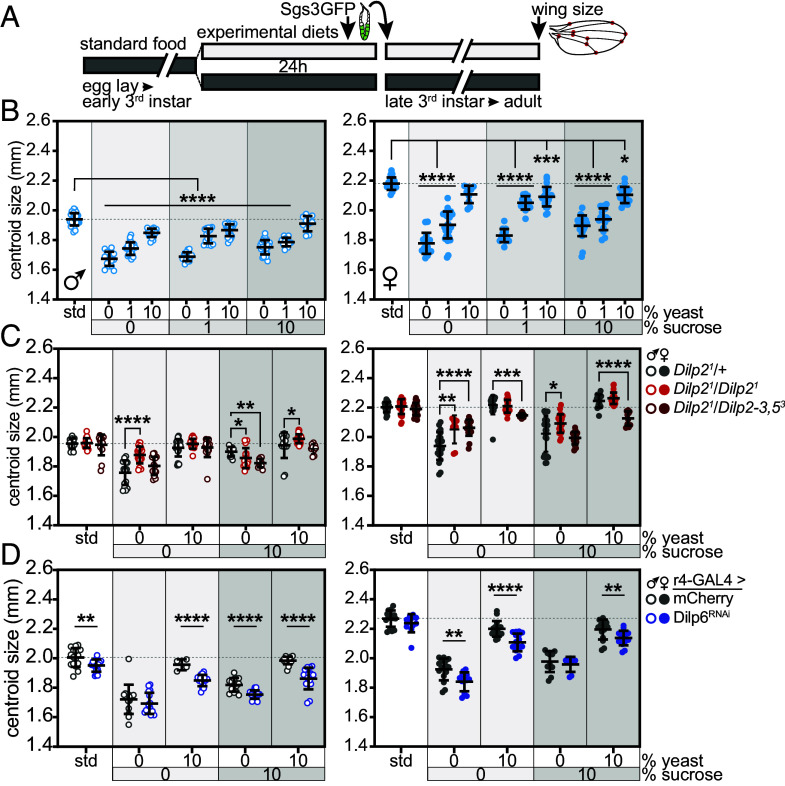
Dietary protein drives peripheral growth. (*A*) Early- to mid-third instar larvae were fed standard food or experimental diets for 24 h, and those with Sgs3-GFP fluorescence indicating progression to ~108 h AEL were transferred to vials containing the same experimental diet for the remainder of the larval and pupal stages. Centroid size was measured in wings dissected from 2 to 3 d old adult flies. (*B*) Wing centroid size in male (*Left*) and female (*Right*) flies carrying *Sgs3-GFP*, n = 10 to 31 wings/group. *P = 0.0110, ***P = 0.0006, ****P < 0.0001 versus standard diet. (*C*) Wing centroid size in adult male (*Left*) and female (*Right*) flies of indicated genotypes, n = 9 to 24 wings/group for each sex. All flies carried *Sgs3-GFP*. *P ≤ 0.0422, **P ≤ 0.0018, ***P = 0.0005, ****P < 0.0001 versus *Dilp2^1^/+*. (*D*) Wing centroid size in adult male (*Left*) and female (*Right*) flies that expressed mCherry or Dilp6^RNAi^ in larval fat body, n = 12 to 16 wings/group for each sex except r4>mCherry males fed 0S/10Y, n = 4 wings and r4>Dilp6^RNAi^ females fed 10S/0Y, n = 8 wings. All flies carried *Sgs3-GFP*. **P ≤ 0.0176 and ****P < 0.0001 versus r4>mCherry. Gray shading in graphs indicates sucrose dose. Data are presented as means ± SD; *P* values were determined by one-way ANOVA with Dunnett’s multiple comparison test and Student’s unpaired *t* tests.

Insulin-like peptides are major endocrine regulators of whole-animal growth in *Drosophila* (4, 5, 14, 16, 43), and protein is the key dietary component for driving growth. Diets with elevated protein to carbohydrate ratios promote whole-animal and wing growth (44, 45). We evaluated wing growth in animals reared on standard food or experimental diets from the mid-third instar (~84 h AEL) through the pupal stage. After 24 h on a given diet, ~108 h AEL larvae (staged with Sgs3-GFP) were selected and returned to their experimental diet. Wing centroid size was measured in adult flies (Fig. 4*A*). Starvation of mid-third instar larvae on the 0S/0Y diet through the pupal stage strongly inhibited wing growth by 14% and 18%, in males and females, respectively, compared with flies fed standard food. Diets containing 10% sucrose alone (10S/0Y) partially rescued growth compared with the 0S/0Y diet, leading to wings that were 10% (males) and 13% (females) smaller than those from animals fed standard food. In contrast, diets containing 10% yeast extract alone (0S/10Y) rescued wing size to 95% (males) and 97% (females) of that in flies fed standard food. The 10S/10Y experimental diet led to a nearly complete rescue of growth compared with the standard diet (Fig. 4*B* and *SI Appendix*, Fig. S5*A*). The presence of the epitope tagged *Dilp2^HF^* or *Dilp6^HF^* alleles led to similar diet-dependent changes in wing size compared with animals carrying *Sgs3-GFP* alone. However, we noted that wing growth responses to the 10S/10Y diet were blunted in *Dilp6^HF^*; *Sgs3-GFP* animals compared with *Dilp2^HF^*; *Sgs3-GFP* animals (*SI Appendix*, Fig. S5 *B* and *C*). We assessed wing imaginal disc mRNA levels of *InR* and *Myc*, a known target of insulin signaling in the wing disc (46, 47). Transcripts encoding *InR* were elevated in wing discs from larvae fed diets low in nutrients, and elevated levels of dietary yeast and/or sucrose normalized *InR* expression (compare 0S/0Y and 0S/10Y; 0S/1Y and 1S/1Y) (*SI Appendix*, Fig. S6*A*). In contrast, *Myc* transcript levels were not strongly regulated by different nutrients (*SI Appendix*, Fig. S6*B*).

Dilp2 and Dilp6 act in an endocrine manner to promote whole-animal growth (4, 5, 12–16). Surprisingly, in *Dilp2^1^* homozygotes, adult wing size was increased relative to *Dilp2^1^* heterozygotes when these animals were reared from the mid-third instar on the 0S/0Y diet. In male *Dilp2^1^* homozygotes, wing size was also increased following rearing on the 10S/10Y diet, while in females homozygous for *Dilp2^1^*, wing size was increased on the 10% sucrose diet (10S/0Y). In animals lacking both alleles of *Dilp2* that were also heterozygous for *Dilp3* and *Dilp5* null alleles, wing size was altered compared with *Dilp2^1^* heterozygotes in a complex sex- and diet-specific manner (Fig. 4*C*). In contrast, animals with fat body-specific knockdown of Dilp6 exhibited reduced wing growth on most diets (Fig. 4*D*). Loss of fat body-derived Dilp6 impaired wing growth even on diets with elevated protein (0S/10Y and 10S/10Y) that led to low circulating Dilp6 levels. This suggests that even low levels of Dilp6 sustain growth, but we acknowledge that Dilp6 is also produced during the pupal phase and that loss of Dilp6 expression in fat body during metamorphosis likely also contributes to the growth defects seen here. Our data reveal some sex differences. On standard food, males were more strongly affected by loss of circulating Dilp6 than females, as observed in animals homozygous for the *Dilp6^41^* null allele (48). Additionally, in animals fed the 10S/10Y diet, males lacking Dilp6 in fat body exhibited a 6.2% decrease in wing size, while females exhibited a 2.7% decrease in wing size compared with controls.

**Fig. 5.**
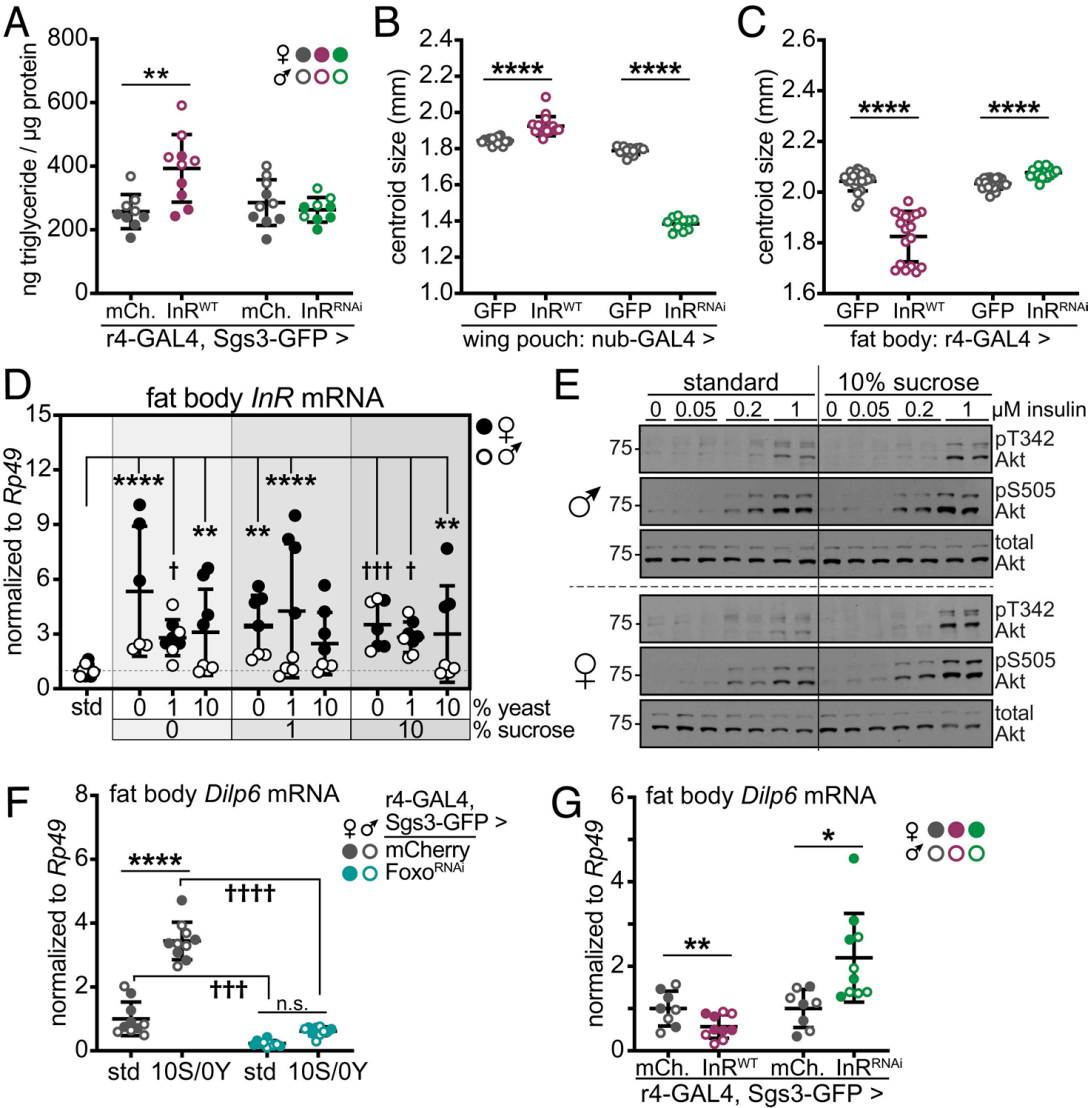
Fat body-derived Dilp6 and fat body insulin signaling exert opposing effects on triglyceride storage and animal growth. (*A*) Whole-animal triglyceride levels, normalized to total protein, in ~108 h AEL larvae fed standard food and carrying *Sgs3-GFP*, *r4-GAL4*, and indicated UAS transgenes, n = 4 to 5 larvae/sex/group, with 9 to 10 total larvae/group. **P = 0.0031 versus r4>mCherry (mCh.). (*B* and *C*) Wing centroid size in adult male flies reared on standard food that expressed indicated UAS transgenes (*B*) in the wing pouch using nubbin-GAL4, n = 10 to 16 wings/group, or (*C*) in larval fat body using r4-GAL4, n = 18 to 23 wings/group. ****P < 0.0001 versus nub>GFP or r4>GFP. (*D*) Fat body transcript levels of *InR* normalized to *Rp49*. n = 3 to 4 samples/sex/group, with 6 to 8 samples total/group. **P ≤ 0.0060, ****P < 0.0001 versus standard diet (females) and †*P* ≤ 0.0446, †††*P* = 0.0003 versus standard diet (males). (*E*) Western blot analysis of phosphorylated (Thr342 and Ser505) and total Akt levels in fat bodies dissected from male (*Top*) and female (*Bottom*) larvae fed standard diet or 10% sucrose and stimulated with human insulin ex vivo at indicated doses for 15 min. (*F* and *G*) Fat body transcript levels of *Dilp6*, normalized to *Rp49*, in ~108 h AEL larvae fed standard food or the 10S/0Y diet for 24 h and carrying *Sgs3-GFP*, *r4-GAL4*, and indicated UAS transgenes, n = 4 to 5 samples/sex/group, with 8 to 10 samples total/group. (*F*) ****P < 0.0001 versus standard diet; †††*P* = 0.0007 and ††††*P* < 0.0001 versus r4>mCherry. G: *P = 0.0191, **P = 0.0083 versus r4>mCherry. For panels *A*–*D*, *F*, and *G*, female and male samples are indicated by filled and open symbols, respectively. Data are presented as means ± SD; *P* values were determined by Student’s unpaired *t* tests and one-way ANOVA with Dunnett’s multiple comparison test.

Our data raise the question of what roles insulin signaling plays in nutrient storage and growth, downstream of Dilps. To address this, we manipulated InR in fat body using r4-GAL4, collected larvae at ~108 h AEL, and assessed triglyceride storage in animals fed the standard diet. Elevated expression of wild type InR in fat body led to increased triglyceride levels, while knockdown of InR had no effect on triglyceride storage (Fig. 5*A*). Next, we examined cell-autonomous and cell-nonautonomous roles of InR in wing growth. We overexpressed or knocked down InR in the wing imaginal disc pouch using nubbin-GAL4. Elevated expression of wild type InR increased wing size by 4.3% compared with controls expressing GFP, while knockdown of InR decreased wing size by 25% (Fig. 5*B* and *SI Appendix*, Fig. S7*A*), consistent with known, cell-autonomous roles of insulin signaling in driving wing growth (3, 4, 6, 49, 50). We then manipulated InR levels in larval fat body using r4-GAL4. Surprisingly, elevated expression of wild type InR in fat body led to a 21% decrease in wing size compared with controls expressing GFP. In contrast, knocking down InR in fat body led to a 2.1% increase in wing size compared with controls (Fig. 5*C* and *SI Appendix*, Fig. S7*B*).

Finally, we asked whether insulin signaling in fat body regulates *Dilp6*, given that both *InR* and *Dilp6* are positively regulated by Foxo (16, 51), a transcription factor inhibited by insulin signaling (52). First, we assessed *InR* mRNA levels and phosphorylation of the downstream kinase Akt in larvae fed standard food and high sucrose, diets that promote *Dilp6* expression. Fat body *InR* expression was increased by most experimental diets, with stronger effects in females than in males (Fig. 5*D*). *InR* transcript levels were increased 3.2- to 3.9-fold in larvae fed the high sucrose 10S/0Y diet for 24 h. This prompted us to ask whether fat bodies from larvae fed high sucrose were resistant or sensitive to insulin, as previous analyses showed that prolonged high sucrose feeding leads to whole-animal insulin resistance in *Drosophila* larvae (53, 54). We dissected fat bodies from larvae fed standard or 10S/0Y diets for 24 h and stimulated them ex vivo with increasing doses of human insulin. Basal phosphorylation of Akt on Thr342 and Ser505 was low in fat bodies treated with vehicle and increased in both diet conditions in response to increasing doses of insulin (Fig. 5*E* and *SI Appendix*, Fig. S7*C*), indicating that short-term high sucrose feeding does not lead to insulin resistance in fat body. However, Dilp2 levels are low in the 10S/0Y condition, suggesting that Foxo may be active in vivo. Foxo is a major regulator of *Dilp6* expression in fat body, and knockdown of Foxo led to decreased fat body *Dilp6* mRNA in larvae fed standard or 10S/0Y diets (Fig. 5*F* and *SI Appendix*, Fig. S7*D*). Finally, transcript levels of *Dilp6* were decreased by 42% in fat bodies overexpressing wild type InR but increased 2.2-fold in fat bodies with InR knockdown compared with controls (Fig. 5*G*). Consequently, impaired wing growth in animals with elevated fat body InR may be due, at least in part, to reduced Dilp6 production, and increased Dilp6 may underlie increased wing growth in animals with fat body-specific InR knockdown.

## Discussion

Here, we used a nutritional geometry approach to determine how dietary sugar and yeast regulate circulating levels of the InR ligands Dilp2 and Dilp6 in fruit fly larvae, a stage of life characterized by rapid growth and nutrient storage. We found that Dilp2 is sensitive to starvation, and that circulating Dilp2 levels are positively regulated by dietary yeast but not sucrose, in agreement with previous findings assessing Dilp2 retention or release from brain IPCs (18–24). In contrast, Dilp6 was relatively insensitive to starvation, and it was positively regulated by sucrose but, unexpectedly, negatively regulated by dietary yeast. Our data also show that animal growth, like Dilp2, was highly responsive to dietary protein and that nutrient storage strongly correlated with dietary sucrose, much like Dilp6. Loss of Dilp2 had modest, diet-dependent effects on lipid storage and wing growth. Eliminating Dilp6 expression in fat body reduced adult wing size, even when larvae were fed diets high in yeast that suppressed Dilp6 production during the third instar. It is important to note that our experiments do not address roles of Dilp6 in promoting growth during metamorphosis, and whether effects of larval diets on Dilp6 persist in the pupal stage is unknown.

Our results highlight important and distinct roles for dietary sugar and yeast in *Drosophila* larvae. Dietary sugar increased hemolymph trehalose and drove nutrient storage, as previously observed (54). Sucrose promoted growth in the absence of dietary yeast, albeit weakly. Effects on nutrient storage, Dilp6 levels, and growth distinguish diets containing sucrose alone from the 0S/0Y diet that lacks nutrients altogether. This is important because sucrose is often added to minimal media in experiments examining effects of starvation on fruit fly larvae or cultured organs (20, 55). Yeast is the primary protein source for *Drosophila* larvae, and our data show that in the absence of dietary sucrose, dietary yeast drove growth. Compared with males, growth in females was more sensitive both to the lack of dietary yeast and to the rescuing effects of dietary protein. We found increased circulating Dilp2 in females fed diets high in protein compared with males; this likely contributes to greater activity of the insulin signaling pathway in females fed diets high in yeast (56). At low sugar levels, dietary yeast supported triglyceride storage, perhaps due to the small amount of carbohydrate available in yeast extract and/or conversion of dietary amino acids to glucose.

Insulin and related hormones communicate nutrient status between organs, allowing animals to coordinate growth with nutrient availability. In *Drosophila*, loss of insulin signaling pathway components in wing imaginal discs produces tissue-autonomous growth defects (3, 4, 6, 49, 50), a finding replicated here with InR manipulations in the wing pouch. In contrast, manipulations of InR in fat body led to unexpected effects. As an upstream regulator of mechanistic target of rapamycin complex 1 (mTORC1), insulin signaling in fat body might be expected to drive growth of the whole-animal, because mTORC1 acts in the fat body to promote Dilp secretion from brain IPCs (20). However, our data show that loss of InR in fat body promoted animal growth, while increased levels of wild type InR in fat body suppressed growth. We found that increased InR levels in fat body reduced *Dilp6* transcripts, likely accounting for some of the reduced peripheral growth in these animals. However, elevated fat body insulin signaling more strongly suppressed wing growth than loss of Dilp6 from fat body, suggesting that fat body insulin signaling regulates not only Dilp6 but also other factors to control animal growth. Elevated fat body InR levels also led to increased lipid storage. However, knocking down InR did not alter triglyceride stores, suggesting that substrate availability and/or basal activity of downstream pathway components are sufficient for normal nutrient storage in fat body. Indeed, our Western blot data show that insulin signaling in the fat body in vivo is submaximal, as observed in other contexts (41), and likely rarely achieves the level of activity induced by forced expression of InR.

Our data, along with those of other labs, suggest that Dilp6 integrates internal and external stimuli to control growth and metabolism. Dilp6 expression is positively regulated by ecdysone, and Dilp6 levels rise rapidly as animals enter the second half of the third instar (14–17). Our data show that Dilp6 is positively regulated by dietary sugar and negatively regulated by dietary yeast and that circulating levels of Dilp6 are responsive to the ratio of these nutrients, independently of changes in circulating ecdysteroids. The mechanisms for nutrient-specific regulation of Dilp6 are unknown, and it will be of interest to determine whether Dilp6 is regulated not only by Foxo but also by glucose-responsive transcription factors (57) and kinases such as mTORC1 and/or Gcn2 that respond to amino acid availability (58, 59). Finally, we found that insulin signaling in fat body negatively regulates *Dilp6* expression; this likely occurs through Foxo, a key activator of *Dilp6* (16). Innate immune signaling acts cell-autonomously to disrupt insulin signaling in the fat body (60, 61), yet infection and Toll signaling suppress Dilp6 production (15), likely via Toll pathway transcription factors that may predominate over Foxo to regulate *Dilp6*. Whether Dilp6 expression in fat body is regulated by other environmental stimuli, internal factors like specific Dilp(s) acting on the fat body InR, or other hormones remains to be discovered.

How do Dilp2 and Dilp6 coordinate growth and metabolism? Dilp2 circulates in hemolymph until the late stage of the third instar, with levels declining as Dilp6 levels increase. These changes occur as larvae approach the pupal stage (14–17), when triglyceride storage and whole-animal growth have plateaued (2, 15, 17) but as growth of wing imaginal discs continues (62). Our study shows that Dilp2 and Dilp6 exhibit divergent responses to dietary sugar and protein. Dilp6 positively regulates wing growth on a variety of diets, suggesting that it acts as an InR agonist in organs like wing imaginal discs. Loss of Dilp2 had inconsistent effects on wing size, paradoxically leading to increased wing growth on the 0S/0Y diet that lacks nutrients (0S/0Y). Our data show that deletion of *Dilp2* leads to increased Dilp6 levels in hemolymph, while Dilp6 promotes Dilp2 secretion, possibly via paracrine interactions in the brain. Therefore, it is possible that increased wing growth during starvation in *Dilp2* mutants may be due to increased circulating Dilp6. Our data show that fat body-derived Dilp6 antagonizes triglyceride storage in larvae fed a variety of experimental diets, while loss of Dilp2 leads to increased triglyceride levels in larvae fed diets high in sugar. Loss of Dilp2 also leads to increased whole-body trehalose levels (63). Elevated expression of Dilp6 in the pupal fat body leads to increased adult size (14, 16), and Dilp6 promotes lipid turnover (64) and increases metabolic rate concomitant with decreased respiratory quotient (65), indicating that Dilp6 may promote lipid oxidation to support growth and starvation resistance. Taken together, our data and that of other labs suggest that Dilp6 may also direct larvae to use dietary sugars for growth instead of nutrient storage. In this way, Dilp6 may prevent the misdirection of resources, especially at the end of the larval stage as developmental time for nutrient intake becomes limited and local nutrient resources may be depleted. Our data are consistent with this model (*SI Appendix*, Fig. S8).

Data from this study and others raise a key question: How does the single InR encoded in the *Drosophila* genome interpret the information carried by distinct Dilps? One possibility is that InR simply responds to the total level of Dilps in circulation. This seems unlikely given the large differences in hemolymph Dilp2 and Dilp6 in larvae fed 10% yeast or 10% sucrose and the corresponding effects of these diets on growth and metabolism. Insulin-like growth factor (IGF) binding proteins with different affinities for Dilp2 and Dilp6 may act to restrict or promote local access of these hormones to InR, as has been shown for Dilp2 and Dilp5 in the corpora cardiaca (66). Another possibility is that alternative splicing of InR or the presence of additional, unknown coreceptors in Dilp target tissues might bias InR sensitivity to one Dilp over another. This scenario would most closely resemble the condition in mammals, where insulin and IGFs bind to distinct receptors to control downstream processes (67). Finally, it is likely that InR adopts distinct conformations in response to sequential binding of Dilps, alone or in combination. Indeed, altered conformations of human InR are observed as it becomes saturated with insulin and when insulin versus IGF-2 is bound (68, 69). Solving additional structures of *Drosophila* InR bound to individual Dilps will help to address this question (70). Distinct conformations of Dilp-bound InR may lead to differential downstream signaling, as has been observed for insect and mammalian InRs bound by distinct ligands (71–73).

Our findings show that Dilp2 and Dilp6 exhibit divergent responses to the most critical component of an animal’s environment: availability of key dietary nutrients. The exact information carried by each hormone and the targets of its actions remain to be discovered. Nonetheless, multiple Dilps that are regulated by distinct environmental and internal stimuli likely provide flexibility in the *Drosophila* insulin signaling pathway. In the wild, environmental and physiological conditions can change rapidly, and it is possible that the specific Dilps produced in response to these conditions control different aspects of biology, thereby helping the animal adapt and survive.

## Materials and Methods

### *Drosophila* Diets.

Flies were raised on standard food (prepared by Archon Scientific, Durham, NC) containing 9.69% (liquid weight) unsulfured golden molasses (Sweet Harvest, Cannon Falls, MN), 2.42% dried whole yeast (*Saccharomyces cerevisiae*) (Red Star Yeast Co., Milwaukee, WI), 4.58% enriched and degerminated yellow cornmeal (Quaker, Chicago, IL), 0.3% propionic acid, and 0.1% methylparaben. For experimental diets, sucrose (Fisher BP220) or *S. cerevisiae* yeast extract (Fisher BP9727) were added at 1% or 10% concentrations to 0.8% agar (Fisher BP1423). The lots of yeast extract used in the experimental diets contain 11.3% nitrogen (lot 137032) and 11.52% nitrogen (lot 242149). A nitrogen-to-protein conversion factor of 5.62 was used to calculate total protein, in agreement with published data (28–30). *S. cerevisiae* yeast extract is estimated to contain 2.90 to 3.06% carbohydrate (28, 29).

### *Drosophila* Stocks and Husbandry.

Experiments were performed using larvae that were in the early- to mid-third instar stage (~80 to 90 h AEL) at the beginning of each feeding regimen. For all experiments, larvae were floated off food with 2 M NaCl, rinsed in phosphate-buffered saline (PBS), and transferred to standard food or experimental diets for 24 h at a density of 12 to 24 larvae/well in 12-well plates containing 1.5 mL food/well and capped with foam stoppers. For staging using Sgs3-GFP, larvae were gently transferred from experimental diets to PBS, and salivary gland Sgs3-GFP fluorescence was evaluated as described (17). Larvae at ~108 h AEL were collected, sorted for sex, and used for further analyses. The following stocks were obtained from the Bloomington *Drosophila* Stock Center (Bloomington, IN): *Dilp2^1^* (#30881), *Df(3L)Dilp2-3*, *Dilp5^3^* (#30889), Sgs3-GFP (#5885), UAS-Dilp6^RNAi^ (#33684), UAS-InR^WT^ (#8262), UAS-InR^RNAi^ (#51518), UAS-Foxo^RNAi^ (#32427), UAS-mCherry (#38425), UAS-GFP (#1521), r4-GAL4 (#33832), and nubbin-GAL4 (#25754). Other flies used were *Dilp2^1^*, *gDilp2^HF^* (35), *Dilp6^HF^* (15), and *Dilp6^68^* (5).

### Measurement of Food Intake.

Consumption of diets containing blue food dye was measured using ultraviolet-visible spectroscopy (74, 75). Larvae carrying Sgs3-GFP were reared on standard food until the first half of the third instar and then transferred in groups of 20 to 30 larvae per well to 12-well plates containing diets with 1% FD&C Blue No. 1 (0.026 M, McCormick). Larvae were fed blue-dyed food for 24 h or for periods corresponding to hours 0 to 4, 8 to 12, or 20 to 24 of the 24 h feeding period. For the 8 to 12 and 20 to 24 h groups, larvae were transferred from undyed food to wells with the same diet containing 1% FD&C Blue No. 1. Larvae were staged using Sgs3-GFP, and animals that had reached an equivalent developmental stage (~108 h AEL) were used for food consumption measurements. Animals in the 0 to 4 h group exhibited no GFP expression in salivary glands and were ~84 to 92 h AEL. For food intake measurements, larvae were removed from food, rinsed in PBS, separated by sex, briefly dried on a Kimwipe, and sonicated in 100 µL H_2_O in groups of three larvae/tube. Supernatants were cleared by centrifugation, and absorbance was measured at 630 nm using an Infinite 200 PRO plate reader (Tecan). A linear calibration of known FD&C Blue No. 1 amounts was generated and used to calculate the mass of food in the gut of each larva at the time of sample collection.

### Measurement of Hemolymph Dilp Levels by ELISA.

Hemolymph Dilp levels were measured in pooled samples collected on ice from 5 to 6 larvae. 1 µL of pooled hemolymph was used for Dilp2^HF^ and Dilp6^HF^ measurement by ELISA using previously established protocols (15, 35). Briefly, for ELISA measurements, Immuno Clear plates (Thermo Fisher Scientific) were coated overnight at 4 °C with anti-FLAG antibody (Sigma-Aldrich, F1804) diluted to 5 µg/mL in 0.2 M sodium carbonate/bicarbonate buffer (pH 9.4). Plates were washed with PBS containing 0.2% Tween 20 (PBS-T) and blocked in sterile-filtered 2% bovine serum albumin in PBS for 3 h at room temperature. Following washes with PBS-T, FLAG(GS)HA peptide standard (DYKDDDDKGGGGSYPYDVPDYA-amide, 2,412 daltons, used at 0.5 to 50 pg/µL) or hemolymph (diluted 1:100 in ice-cold PBS) was added to each well. Anti-HA-Peroxidase 3F10 antibody (Sigma-Aldrich 12013819001), diluted to 15 ng/mL in PBS with 1% Triton X-100, was added to each well for a final volume of 50 µL/well. Plates were incubated in humidified chambers overnight at 4 °C. After washes with PBS-T, 1-Step Ultra TMB ELISA Substrate (Thermo Fisher Scientific) was added to each well. Plates were incubated for 15 min at room temperature, and reactions were stopped by adding 2 M sulfuric acid. Absorbance was measured at 450 nm using an Infinite 200 PRO plate reader (Tecan). To convert molar concentrations to pg/µL hemolymph, we used molecular weights of 7,828.86 daltons and 10,303.62 daltons for mature Dilp2^HF^ and Dilp6^HF^ proteins, respectively.

### Measurement of Hemolymph Ecdysteroids by ELISA.

Hemolymph ecdysteroids were measured in pooled samples collected on ice from 9 to 10 larvae staged to ~108 h AEL using Sgs3-GFP, as previously described (76). Two µL of pooled hemolymph was mixed with 200 µL of chilled methanol, vortexed for 30 s, and centrifuged at 10,000 rpm for 10 min at 4 °C. The supernatant was carefully transferred to a new tube and dried completely using a nitrogen-blowing sample concentrator. The dried sample was resuspended with 100 µL 1× ELISA assay buffer. Hemolymph ecdysteroid levels were measured using the 20-Hydroxyecdysone Competitive ELISA Kit (Thermo Fisher Scientific, MA, Cat# EIAHYD), following the manufacturer’s protocol.

### Measurement of Hemolymph Sugars.

Hemolymph trehalose and glucose levels were measured in pooled samples collected on ice from 5 to 8 larvae. Endogenous trehalase was destroyed by heating hemolymph diluted in PBS at 70 °C for 20 min. The sample was split in half, 1 mU trehalase (Sigma-Aldrich, T8778) was added to one tube, and both were incubated at 37 °C for 2 h. Glucose in both samples was measured using an Amplex UltraRed assay as described below for glycogen measurements. Trehalose was calculated by subtracting glucose values in trehalase-free samples from glucose values in trehalase-treated samples and then dividing by two as trehalose is a dimer of glucose.

### Measurement of Glycogen and Triglyceride Levels.

For glycogen measurements, individual larvae were homogenized in ice-cold 0.1 M NaOH using a Kontes pestle, heated at 70 °C for 20 min, and cleared by centrifugation at 4 °C. Samples were incubated for 1 h at 37 °C with 0.2 M sodium acetate, pH 4.8, with or without amyloglucosidase (AMG, 5 mg/mL, Sigma-Aldrich 10115). Samples were then incubated for 15 min at 25 °C with assay buffer containing glucose oxidase (0.2 U/mL, Sigma-Aldrich G7141), horseradish peroxidase (0.3 U/mL, Sigma-Aldrich P8250), and Amplex UltraRed (25 µM, Invitrogen A36006). Fluorescence in glucose standards (Fisher CAS 50-99-7) and experimental samples was measured with a Tecan Infinite 200 PRO plate reader (excitation/emission maxima = 535/587 nm). Glycogen levels were calculated by subtracting free glucose (without AMG added) from the amount of AMG-hydrolyzed glucose and then normalized to protein levels (Bicinchoninic (BCA) Protein assay, Pierce). For triglyceride measurements, individual larvae were sonicated in ice-cold buffer containing 140 mM NaCl, 50 mM Tris-HCl, pH 7.4, 0.1% Triton X-100 with protease inhibitors (Roche). Following clearing by centrifugation at 4 °C, glycerol and triglyceride levels in each sample were measured using a Serum Triglyceride Determination Kit (Sigma-Aldrich, components F6428, T2449, and G7793) and normalized to protein levels (BCA Protein assay, Pierce).

### Wing Centroid Size.

Wings were dissected from two- to three-day old adult male and female flies and mounted in Gary’s Magic Mount (1.6 g/mL Canada balsam in methyl salicylate). Images of wings were collected with an Olympus MVX10 microscope and DP73 camera using CellSens software (Olympus). FIJI was used to measure x- and y-coordinates of ten landmarks at the intersections of wing veins with each other or the wing margin. Centroid size was calculated as the square root of the sum of squared distances from each landmark to the wing centroid (77).

### Quantitative RT-PCR.

Total RNA was extracted from larval fat bodies (2 to 5 pooled/sample), larval brains (3 to 5 pooled/sample), and larval wing imaginal discs (5 to 6 discs pooled/sample) using the Direct-zol RNA MicroPrep kit (Zymo Research). DNAse-treated total RNA (1 µg) was used to generate complementary DNA (cDNA) using a High-Capacity cDNA Reverse Transcription kit (Thermo Fisher Scientific). Gene expression was measured using gene-specific primers (listed below). Quantitative PCR reactions were performed on 10 to 20 ng cDNA using SYBR Select Master Mix (Thermo Fisher Scientific) with a Bio-Rad CFX Connect Real-Time PCR Detection System. Relative amounts of transcripts were calculated using the comparative Ct method with *Rp49* or *Act5c* as a reference gene (78).

### Primers for RT-qPCR.

Primers are listed 5′ → 3′.

Dilp6-F→ATATGCGTAAGCGGAACGGT

Dilp6-R→GCAAGAGCTCCCTGTAGGTG

Dilp2-F→ACGAGGTGCTGAGTATGGTGTGCG

Dilp2-R→CACTTCGCAGCGGTTCCGATATCG

Dilp3-F→GTCCAGGCCACCATGAAGTT

Dilp3-R→AAGTTCACGGGGTCCAAAGTT

Dilp5-F→TGGACATGCTGAGGGTTGC

Dilp5-R→CCGCCAAGTGGTCCTCATAAT

sun-F→GACTGCCTGGAGAGCTGC

sun-R→GTCCCGTCTTCAAGGACTCG

Gbp2-F→AGCTCGTCCCCAGAATCCTT

Gbp2-R→TACGTGATGACCATGGTGGC

Eip74EF-F→TCCACAATCTGCTTAGCGGC

Eip74EF-R→GACTGGGCGGAAATGAACCT

ImpE1-F→CCAAGCGCTGTTTGTCATCC

ImpE1-R→ACAACTGCTATTGGCCACCTT

InR-F→GCCAATTCAATAGCGGGATAC

InR-R→CTCGCATAGAACGGATTCACC

Myc-F→CCCAATTTGGAGACCCCCTC

Myc-R→CATCAAGTGGCACGAGGGAT

Foxo-F→ACAAGGGCGATTCGAATAGCAG

Foxo-R→CATCCACCAGGATGACTTGC

Rp49-F→CGCTTCAAGGGACAGTATCTG

Rp49-R→AAACGCGGTTCTGCATGA

Act5c-F→GCACCGTCGACCATGAAGAT

Act5c-R→CTCGTCGTACTCCTGCTTGG

### Western Blot Analysis of Insulin Sensitivity.

Fat bodies were dissected from ~108 h AEL larvae carrying Sgs3-GFP that had been fed standard food or 10% sucrose in 0.8% agar for 24 h. Dissected fat bodies (3/sample) were incubated in vehicle (PBS), 0.05, 0.2, or 1 µM human insulin (Sigma-Aldrich, I9278) in a final volume of 500 µL for 15 min at room temperature. Samples were centrifuged, and pelleted fat bodies were sonicated in sample buffer containing 2% sodium dodecyl sulfate (SDS), 60 mM Tris-HCl, pH 6.8, protease inhibitors (Roche), and phosphatase inhibitors. Equal amounts of protein (5 µg, measured by BCA assay, Pierce) were separated by SDS-polyacrylamide gel electrophoresis, proteins were transferred to nitrocellulose, blocked in 3% bovine serum albumin (BSA) in TBS with 0.1% Tween-20 (TBS-T) and blotted overnight at 4 °C with primary antibodies diluted in 1% BSA in TBS-T. Following washes in TBS-T, membranes were incubated with secondary antibodies in 1% milk in TBS-T, washed again, incubated with ECL (Pierce), and exposed to film. Antibodies used were rabbit anti-phosphoThr342-Akt (61), rabbit anti-phosphoSer505-Akt (4054, Cell Signaling Technology), rabbit anti-Akt (4691, Cell Signaling Technology), and goat anti-rabbit HRP (111-035-003, Jackson ImmunoResearch). To quantify phosphorylated Akt levels, FIJI was used to calculate integrated density from scans of films in regions of interest for phospho-T342, phospho-S505, and total Akt. Phospho-Akt values were divided by total Akt values, and data were normalized to fat bodies cultured with vehicle and dissected from animals fed standard food.

### Statistical Analysis.

Statistical parameters including exact sample sizes (with n referring to biological replicates), data plotted (mean ± SD), *P* values, and statistical tests used are reported in figure legends. Statistical analyses were performed using Graphpad Prism 10. Data were analyzed by Student’s unpaired *t* test, one-way ANOVA with the Dunnett multiple comparisons test, or two-way ANOVA with the Tukey multiple comparisons test.

## Supplementary Material

Appendix 01 (PDF)

## Data Availability

Excel files and jpeg data have been deposited in LibraData (10.18130/V3/WCBM91) (79).
