# Supplementary material for: The insulin-like peptides Dilp2 and Dilp6 exhibit divergent responses to dietary sugar and protein in Drosophila larvae
Source: Proc Natl Acad Sci U S A. 2025 Oct 15;122(42):e2426930122. doi: 10.1073/pnas.2426930122 (PMC12557502; doi:10.1073/pnas.2426930122)
Supplement: Supplementary file 1 — Appendix 01 (PDF) [file pnas.2426930122.sapp.pdf]

Supporting information for:

The insulin-like peptides Dilp2 and Dilp6 exhibit divergent responses to dietary sugar and protein in *Drosophila* larvae.

Miyuki Suzawa[1], W. Kyle McPherson[1,2], Kelly E. Dunham[1], Elizabeth E. Van Gorder[1], Shivani Reddy[1,3], Leila A. Jamali[1], and Michelle L. Bland[1,*]

[1]Department of Pharmacology, University of Virginia, Charlottesville, VA 22908-0875

[2,3]Current affiliations: [2]University Program in Genetics & Genomics, Duke University, Durham, NC 27705, [3]University of Alabama School of Medicine, Birmingham, AL 35233

This document contains:
Supplementary Figures S1-S8
Figure Legends for Figures S1-S8

A

g / 100g:	carb.	protein	fat	% in diet
molasses	70	0	0	9.69
cornmeal	74	7.4	0	4.58
yeast	33	52	5	2.42
yeast ext.	3	63.5	0	0, 1, 10
sucrose	100	0	0	0, 1, 10

B

g / 100g:	carb.	protein	fat	kcal	kJ
standard	10.97	1.60	0.12	51.4	213.2
0S 0Y	0	0	0	0	0
0S 1Y	0.03	0.64	0	2.7	11.2
0S 10Y	0.30	6.35	0	26.6	110.3
1S 0Y	1.00	0	0	4.0	16.6
1S 1Y	1.03	0.64	0	6.7	27.8
1S 10Y	1.30	6.35	0	30.6	126.9
10S 0Y	10.00	0	0	40.0	165.9
10S 1Y	10.03	0.64	0	42.7	177.1
10S 10Y	10.30	6.35	0	66.6	276.3

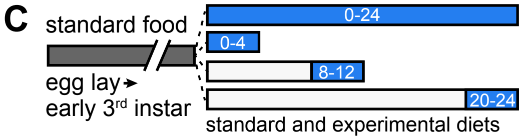

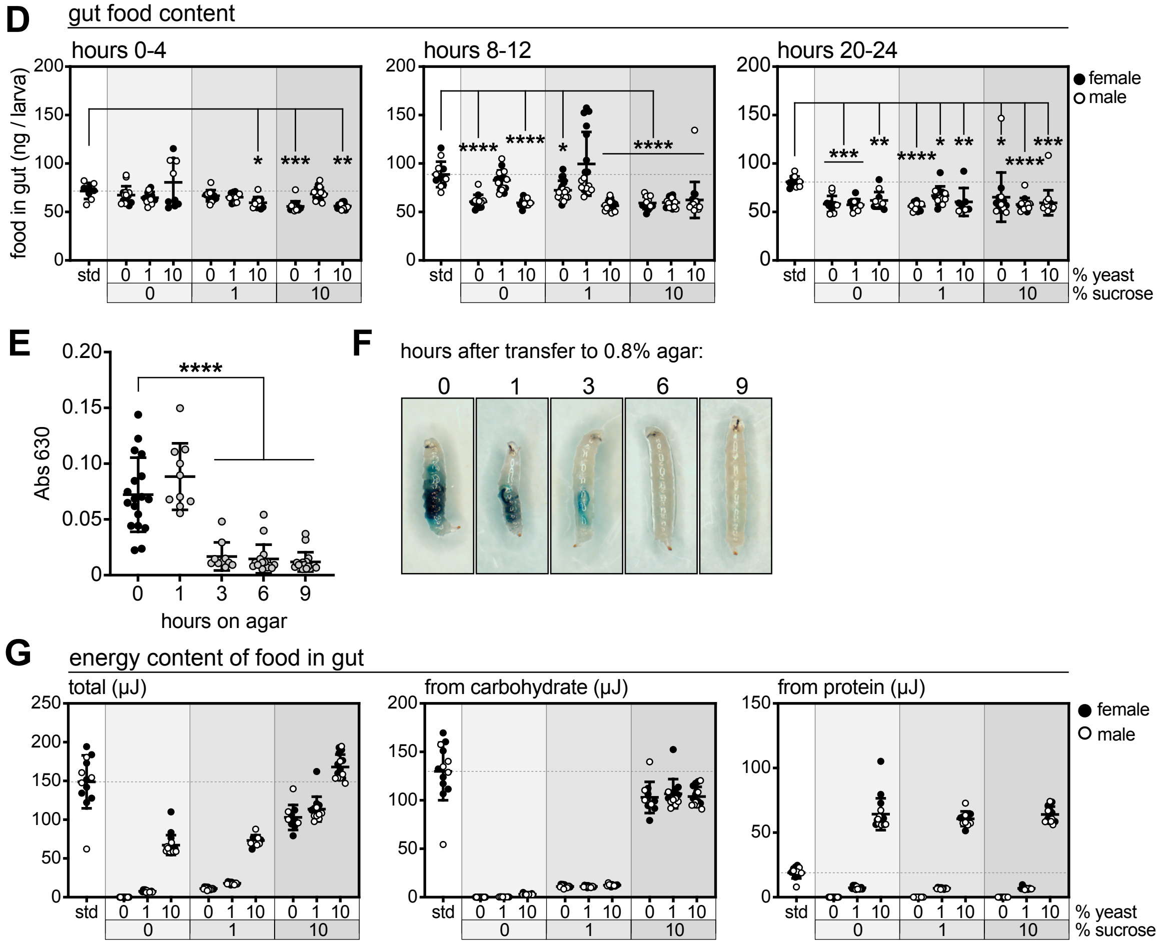

Figure S1. Nutrient composition and caloric values of standard and experimental diets.
A) Carbohydrate, protein, and fat content of individual diet constituents used in this study. Values are from manufacturers' food labels and certificates of analysis. **B)** Carbohydrate, protein, and fat content of standard molasses-cornmeal-yeast fly food and the experimental diets used in this study. Total kcal values were calculated using Atwater general factors (4.0 kcal/g carbohydrate or protein, 9.0 kcal/g fat). **C)** Schematic of experimental approach for determining food intake using diets containing 1% FD&C Blue No. 1. **D)** Amount of food in gut in *Sgs3-GFP* larvae fed standard and experimental diets containing 1% FD&C Blue No.1 for 4-h periods corresponding to the onset (0-4 h, left, n = 3-8 samples/sex/diet, with 8-16 samples total/diet), midpoint (8-12 h, center, n = 5-10 samples/sex/diet, with 12-19 samples total/diet), and end (20-24 h, right, n = 2-10 samples/sex/diet, with 7-18 samples total/diet) of the standard 24 h feeding period used throughout this study. *$p \leq 0.0463$, **p 0.0052, ***$p \leq 0.0007$, ****$p < 0.0001$ versus standard diet. **E)** Mid-third instar larvae reared on standard food containing FD&C Blue No. 1 were transferred to agar for indicated periods of time to monitor progression of ingested food through the gut. Quantitation of blue food in larval lysates, n = 10-18 samples/group. Larvae were not sorted by sex. ****$p < 0.0001$ versus 0 h. **F)** Representative images of larvae at each time point in panel E. **G)** Energetic content of food in gut in *Sgs3-GFP* larvae fed standard and experimental diets containing 1% FD&C Blue No.1 for 24 h, from ~84-108 h AEL, calculated as total energy/gut (left), energy from carbohydrate/-gut (center), and energy from protein/gut (right). n = 4-11 samples/sex/diet, with 10-21 samples total/diet. Gray shading in graphs indicates sucrose dose; female and male samples are indicated by filled and open symbols, respectively. Data are presented as means ± SD; p values were determined by one-way ANOVA with Dunnett's multiple comparison test.

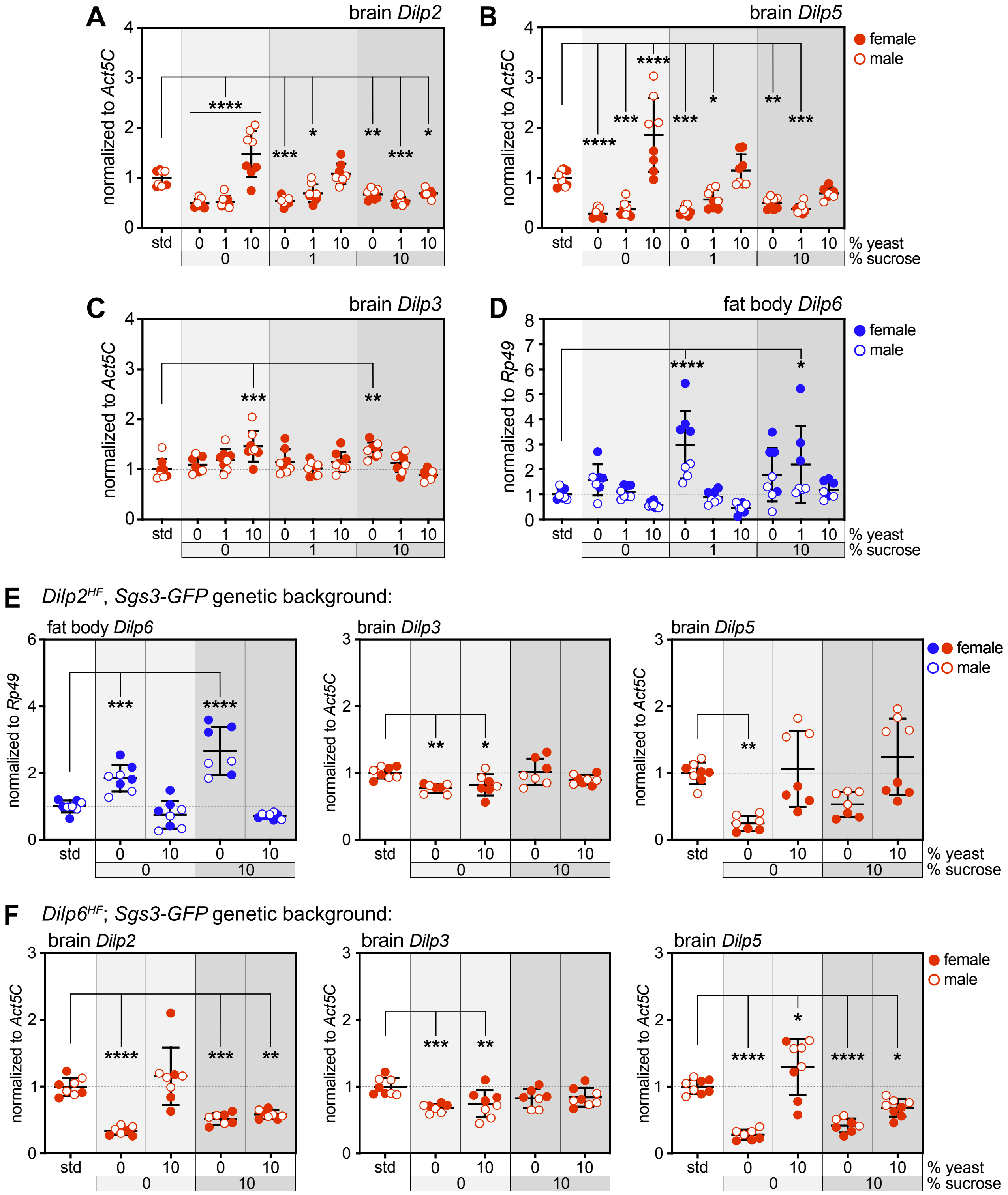

Figure S2. Transcript levels of Dilp2, Dilp3, Dilp5 in brain and Dilp6 in fat body in larvae fed standard and experimental diets. Early- to mid-third instar larvae carrying *Sgs3-GFP* were fed standard and experimental diets for 24 h and collected at ~108 h AEL. **A-C)** Whole-brain transcript levels of: **A)** Dilp2, **B)** Dilp5, and **C)** *Dilp3*, each normalized to *Act5c*. **D)** Fat body transcript levels of *Dilp6*, normalized to *Rp49*. For panels A-D, n = 4 samples/sex/group, with 8 samples total/group. **E)** Fat body transcript levels of *Dilp6*, normalized to *Rp49*, and brain transcript levels of *Dilp3* and *Dilp5*, normalized to *Act5C*, in *Dilp2[1]*, *gDilp2[HF]*, *Sgs3-GFP* larvae. **F)** Brain transcript levels of *Dilp2*, *Dilp3*, and *Dilp5*, normalized to *Act5C*, in *Dilp6[HF]*; *Sgs3-GFP* larvae. For panels E and F, n = 3-4/sex/group with 7-8 samples total/group. *$p \leq 0.0316$, **$p \leq 0.0085$, ***$p \leq 0.0009$, ****$p < 0.0001$ versus standard diet. Gray shading in graphs indicates sucrose dose; female and male samples are indicated by filled and open symbols, respectively. Data are presented as means ± SD; p values were determined by one-way ANOVA with Dunnett's multiple comparison test.

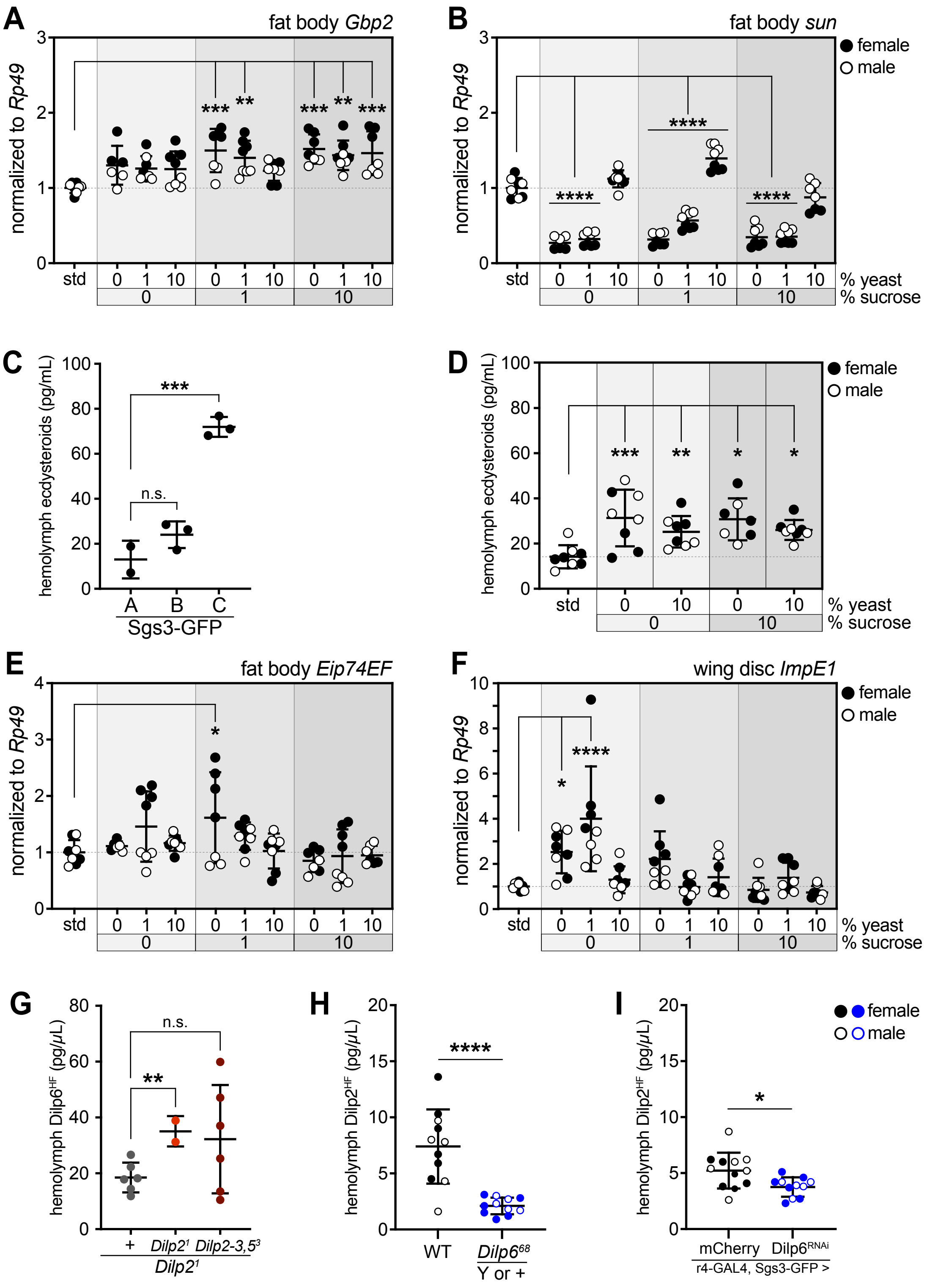
A
fat body Gbp2
normalized to Rp49
B
fat body sun
female
male
std
% yeast
% sucrose
C
hemolymph ecdysteroids (pg/mL)
n.s.
Sgs3-GFP
D
E
fat body Eip74EF
F
wing disc ImpE1
G
hemolymph Dilp6HF (pg/μL)
Dilp2¹
Dilp2-3,5³
H
hemolymph Dilp2HF (pg/μL)
WT
Dilp6⁶⁸
Y or +
I
mCherry
Dilp6RNAi
r4-GAL4, Sgs3-GFP >

Figure S3. Levels of endocrine regulators of Dilp2 and Dilp6 in larvae fed standard and experimental diets. Early- to mid-third instar larvae carrying *Sgs3-GFP* were fed standard and experimental diets for 24 h and collected at ~108 h AEL for further analysis. **A,B)** Fat body transcript levels of (**A**) *Gbp2* and (**B**) *sun*, normalized to *Rp49*. n = 3-4 samples/sex/group, with 6-8 samples total/group. **C)** Hemolymph ecdysteroid levels in *Sgs3-GFP* larvae fed standard food at Sgs3-GFP stages A (~100 h AEL), B (~108 h AEL), and C (~120 h AEL). n = 2-3/group. **D)** Hemolymph ecdysteroid levels in *Sgs3-GFP* larvae fed indicated diets. n = 3-4/sex/group, with 7-8 samples total/group. **E)** Fat body transcript levels of *Eip74EF*, normalized to Rp49. n = 3-4 samples/sex/group, with 6-8 samples total/group. **F)** Wing imaginal disc transcript levels of *ImpE1*, normalized to *Rp49*. n = 4 samples/sex-/group, with 8 samples total/group. For panels A-F, *p ≤ 0.0363, **p ≤ 0.003, ***p ≤ 0.0007, ****p < 0.0001. **G)** Hemolymph Dilp6^{HF} levels in female *Dilp6*^{HF}/+ larvae carrying *Sgs3-GFP* and *Dilp2*^{1}/+ (gray symbols), *Dilp2*^{1}/*Dilp2*^{1} (red symbols), or *Dilp2-3*, *Dilp5*^{3}/*Dilp2*^{1} (dark red symbols), n = 2-6/group. **H)** Hemolymph Dilp2^{HF} levels in *Dilp2*^{1}, *gDilp2*^{HF}, *Sgs3-GFP*/+ larvae with wild type (+/+ females or +/Y males) or *Dilp6*^{68} (*Dilp6*^{68}/+ females and *Dilp6*^{68}/Y males) X chromosomes, n = 5-6 samples/sex/group, with 11 samples total/group. **I)** Hemolymph Dilp2^{HF} levels in *r4-GAL4*, *Dilp2*^{1}, *gDilp2*^{HF}, *Sgs3-GFP*/+ larvae expressing mCherry or Dilp6^{RNAi} in fat body under control of r4-GAL4, n = 5-6/sex group, with 11-12 samples total/group. For panels G-I, *p = 0.0138, **p = 0.0092, ****p < 0.0001 as indicated. Gray shading in graphs indicates sucrose dose; female and male samples are indicated by filled and open symbols, respectively. Data are presented as means ± SD. P values were determined by one-way ANOVA with Dunnett's multiple comparison test or Student's unpaired t tests.

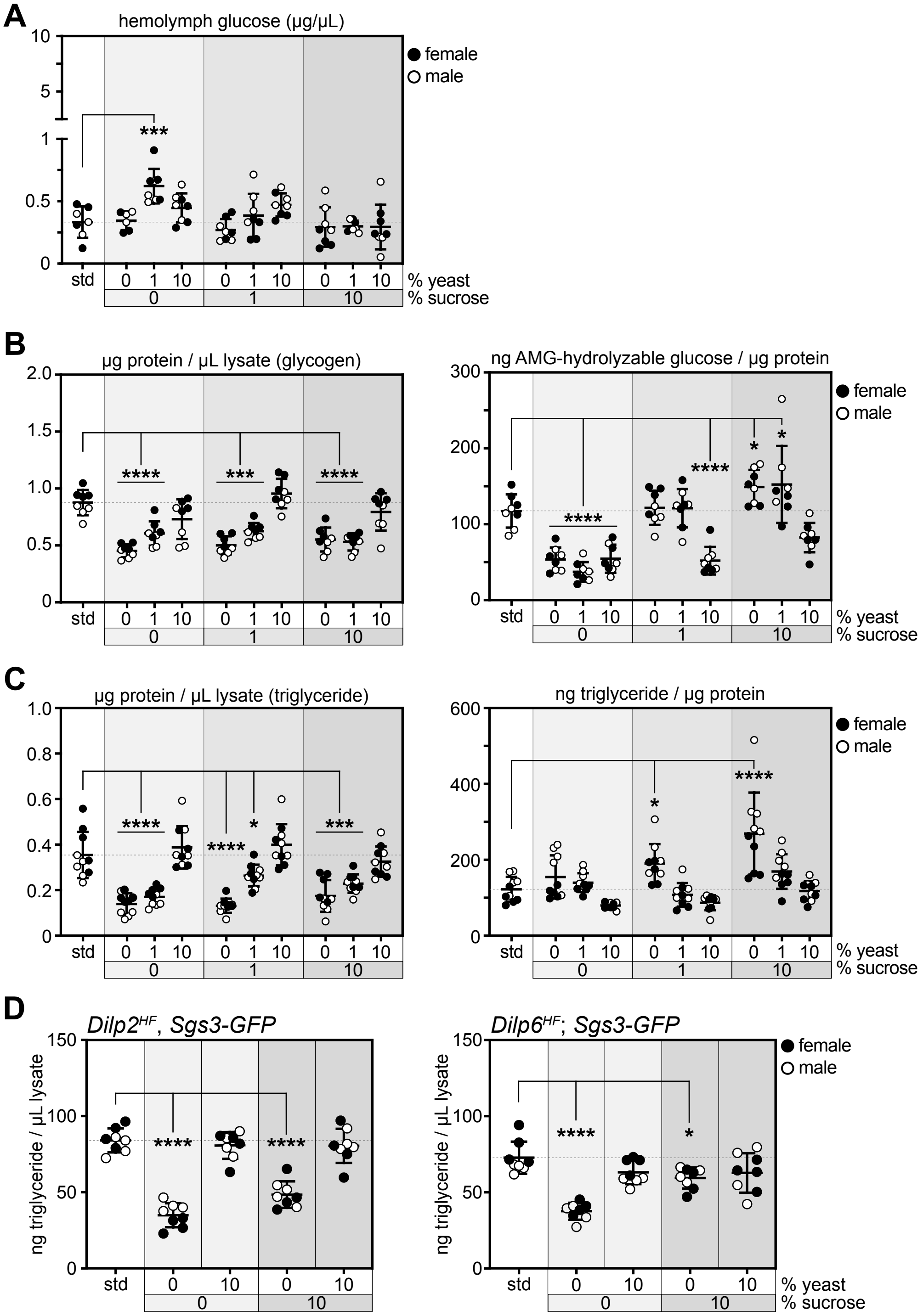

Figure S4. Dietary sugar drives nutrient storage and dietary yeast drives whole-animal protein levels.
Early- to mid-third instar larvae carrying Sgs3-GFP were fed standard food or experimental diets for 24 h, and those with salivary gland Sgs3-GFP fluorescence indicating progression to ~108 h AEL were selected for further analysis. **A)** Hemolymph glucose levels, n = 4 samples/sex/group, with 8 total samples/group. **B)** Left: whole-animal protein levels, normalized to lysate volume, in samples used for glycogen measurement. Right: whole-animal glycogen levels, normalized to whole-animal protein, n = 4 larvae/sex/group with 8 total larvae/group. **C)** Left: whole-animal protein levels, normalized to lysate volume, in samples used for triglyceride measurement. Right: whole-animal triglyceride levels, normalized to whole-animal protein, n = 5 larvae/sex/group with 10 total larvae/group. **D)** Whole-animal triglyceride levels in *Dilp2¹*, *gDilp2^HF*, *Sgs3-GFP* (left) and *Dilp6^HF*; *Sgs3-GFP* (right) larvae, normalized to lysate volume, n = 4 larvae/sex/group, with 8 total larvae/group. For panels A-D, *p ≤ 0.0435, ***p ≤ 0.0005, ****p < 0.0001 versus standard diet. Gray shading in graphs indicates sucrose dose; female and male samples are indicated by filled and open symbols, respectively. Data are presented as means ± SD; p values were determined by one-way ANOVA with Dunnett's multiple comparison test.

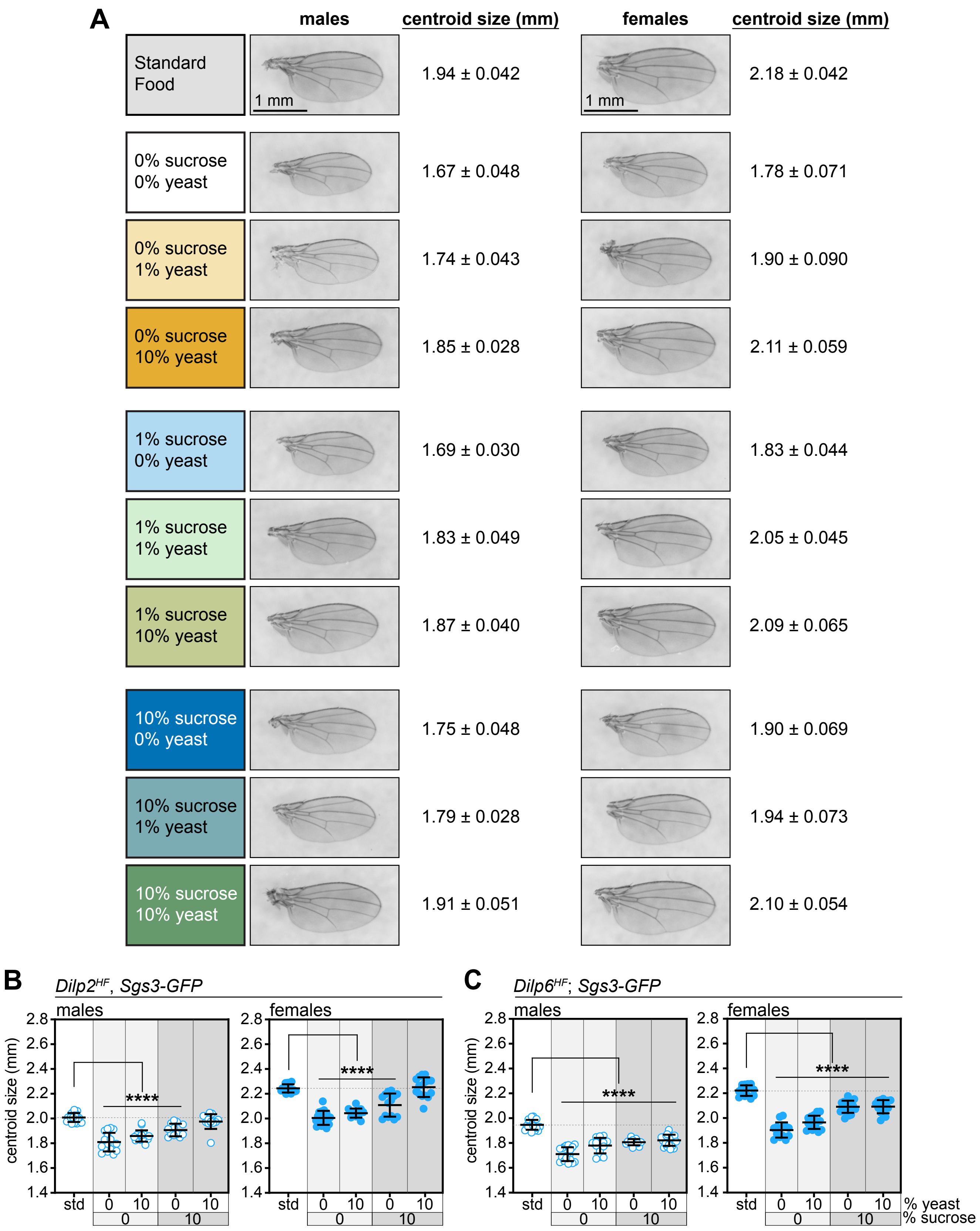
A
males
centroid size (mm)
females
centroid size (mm)
Standard Food
1 mm
1.94 ± 0.042
2.18 ± 0.042
0% sucrose 0% yeast
1.67 ± 0.048
1.78 ± 0.071
0% sucrose 1% yeast
1.74 ± 0.043
1.90 ± 0.090
0% sucrose 10% yeast
1.85 ± 0.028
2.11 ± 0.059
1% sucrose 0% yeast
1.69 ± 0.030
1.83 ± 0.044
1% sucrose 1% yeast
1.83 ± 0.049
2.05 ± 0.045
1% sucrose 10% yeast
1.87 ± 0.040
2.09 ± 0.065
10% sucrose 0% yeast
1.75 ± 0.048
1.90 ± 0.069
10% sucrose 1% yeast
1.79 ± 0.028
1.94 ± 0.073
10% sucrose 10% yeast
1.91 ± 0.051
2.10 ± 0.054
B
Dilp2HF, Sgs3-GFP
C
Dilp6HF; Sgs3-GFP
males
females
centroid size (mm)
****
std
0
10
% yeast
% sucrose

Figure S5. Representative images of wings from flies grown on experimental diets.
A) Wings were dissected from 2-3 day old adult male and female flies carrying *Sgs3-GFP* that were grown on each of ten diets for 24 hours from the early- to mid-third instar stage, selected for Sgs3-GFP fluorescence indicating progression to ~108 h AEL, and transferred to vials containing the same diet for the remainder of the larval and pupal stages. Representative images were chosen based on centroid values closest to the experimental mean of each group, shown to the right of each image. Scale bar, 1 mm. **B,C)** Adult wing centroid size in male (left) and female (right) flies, reared on standard food or experimental diets from mid-third instar to eclosion. (**B**) *Dilp2*^{1}, *gDilp2*^{HF}, *Sgs3-GFP* animals, n = 12-20 wings/group. (**C**) *Dilp6*^{HF}; *Sgs3-GFP* animals, n = 14-20 wings/group. ****$p < 0.0001$ versus standard diet. Gray shading in graphs indicates sucrose dose. Data are presented as means ± SD; p values were determined by one-way ANOVA with Dunnett's multiple comparison test.

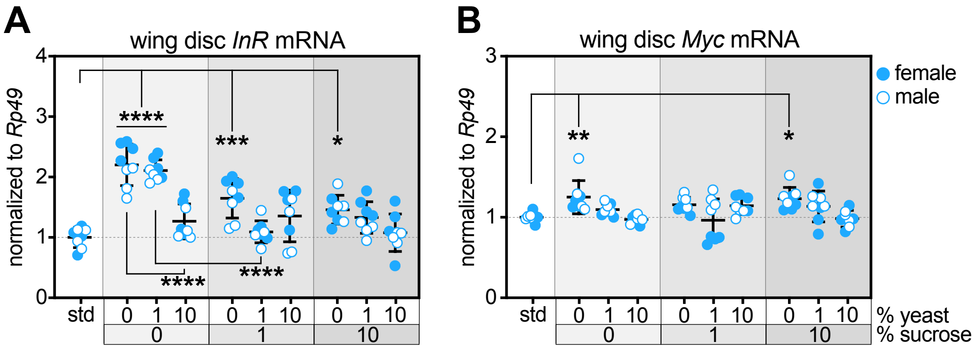

Figure S6. Wing disc-specific expression of *InR* and *Myc* in larvae fed experimental diets.
A,B) Wing imaginal disc transcript levels of (**A**) *InR* and (**B**) *Myc*, normalized to *Rp49*. n = 4 samples/sex/group, with 8 samples total/group. *p ≤ 0.0150, **p = 0.0074, ***p = 0.0002, ****p < 0.0001. Gray shading in graphs indicates sucrose dose; female and male samples are indicated by filled and open symbols, respectively. Data are presented as means ± SD; p values were determined by one-way ANOVA with Dunnett's multiple comparison test and Student's unpaired t test (pairwise comparisons in panel A).

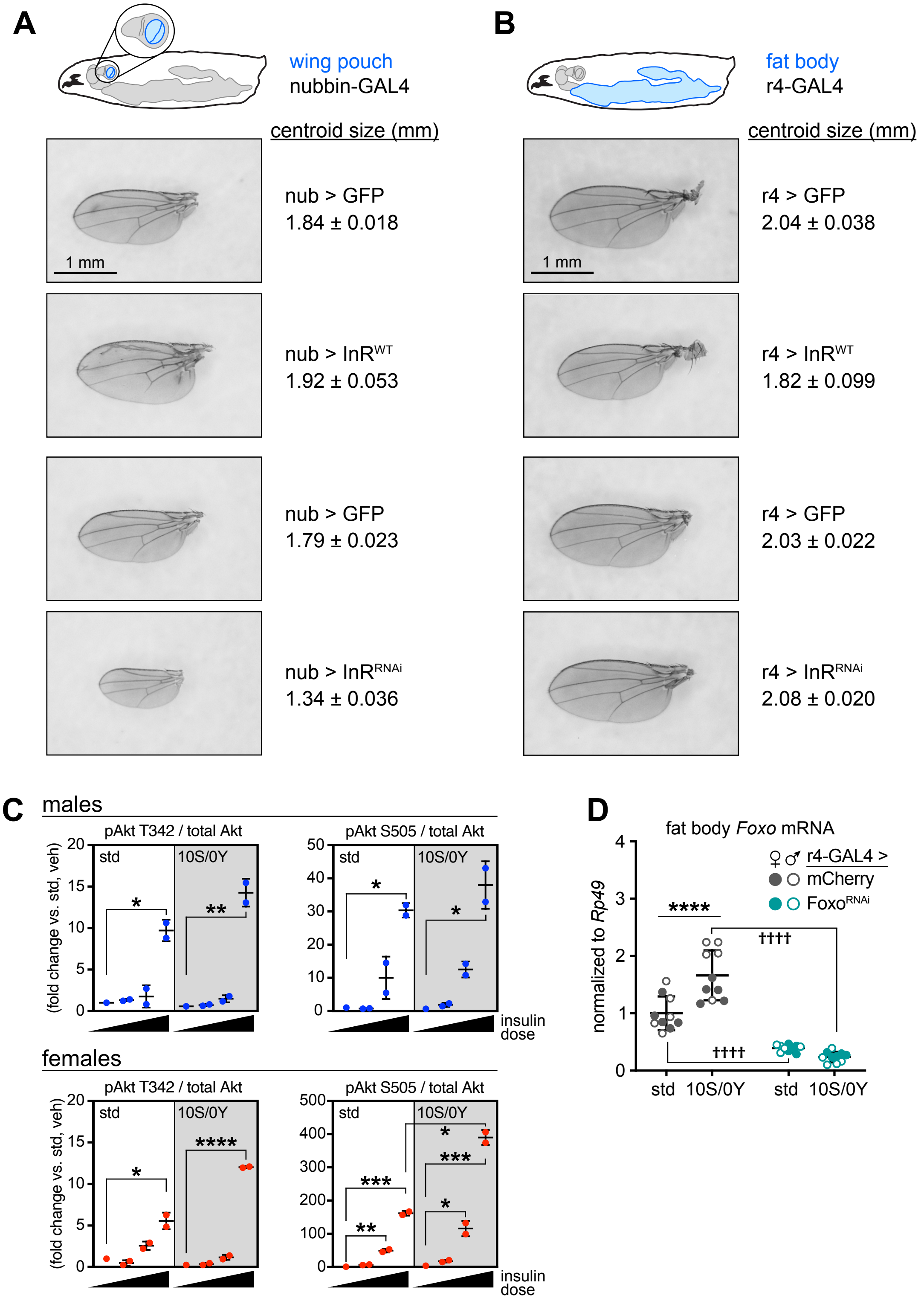
A
wing pouch
nubbin-GAL4
centroid size (mm)
1 mm
nub > GFP
1.84 ± 0.018
nub > InR^WT
1.92 ± 0.053
nub > GFP
1.79 ± 0.023
nub > InR^RNAi
1.34 ± 0.036
B
fat body
r4-GAL4
centroid size (mm)
1 mm
r4 > GFP
2.04 ± 0.038
r4 > InR^WT
1.82 ± 0.099
r4 > GFP
2.03 ± 0.022
r4 > InR^RNAi
2.08 ± 0.020
C
males
pAkt T342 / total Akt
pAkt S505 / total Akt
(fold change vs. std, veh)
std
10S/0Y
insulin dose
females
pAkt T342 / total Akt
pAkt S505 / total Akt
(fold change vs. std, veh)
std
10S/0Y
insulin dose
D
fat body Foxo mRNA
normalized to Rp49
♀ ♂ r4-GAL4 >
mCherry
Foxo^RNAi
****
††††
††††
std
10S/0Y
std
10S/0Y

Figure S7. Representative images of wings from flies with wing disc and fat body InR manipulations. **A)** Top: schematic showing expression domain of nubbin-GAL4 in the imaginal wing disc pouch (blue). Bottom: representative images of wings from flies expressing UAS-GFP, UAS-InR^{WT} or UAS-InR^{RNAi} in the wing pouch. **B)** Top: schematic showing expression domain of r4-GAL4 in the larval fat body (blue). Bottom: representative images of wings from flies expressing UAS-GFP, UAS-InR^{WT} or UAS-InR^{RNAi} in the fat body. Centroid sizes were measured in wings dissected from 2-3 day old adult male flies that were grown on the standard diet. Representative images were chosen based on centroid values closest to the experimental mean of each group, shown to the right of each image. Scale bar, 1 mm. **C)** Quantitation of Western blots of phosphorylated and total Akt in fat bodies, shown in Figure 5E. All data were normalized to values obtained from fat bodies cultured with vehicle and dissected from animals fed standard diet, n = 2 samples per insulin dose except vehicle, n = 1, * $p \leq 0.0318$, **$p \leq 0.0088$, ***$p \leq 0.0009$, ****$p < 0.0001$, as indicated. **D)** Fat body transcript levels of *Foxo*, normalized to *Rp49*, in ~108 h AEL larvae fed standard food or the 10S/0Y diet for 24h and carrying *Sgs3-GFP*, *r4-GAL4*, and indicated UAS transgenes, n = 5 samples/sex/group, with 10 samples total/group. ****$p < 0.0001$ versus standard diet; ††††$p < 0.0001$ versus r4>mCherry, female and male samples are indicated by filled and open symbols, respectively. Data are presented as means ± SD; p values were determined by Student's unpaired t tests and one-way ANOVA with Dunnett's multiple comparison test.

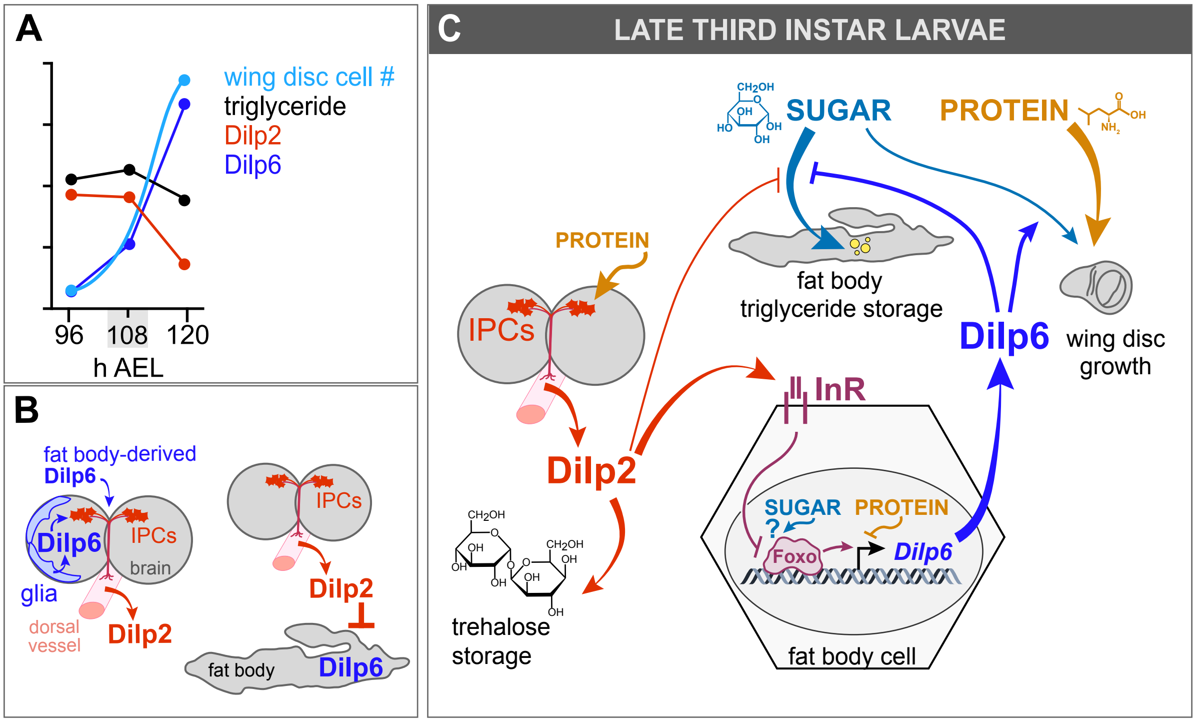

Figure S8. Model of regulation of growth and nutrient storage by Dilp2 and Dilp6 in *Drosophila* third instar larvae.

A) Schematic of developmental regulation of wing disc growth, triglyceride storage, and hemolymph levels of Dilp2 and Dilp6 during the final 24 hours of the third larval instar (96-120 h AEL). Most measurements in this study were made in larvae at ~108 h AEL. **B)** Left: Dilp6 promotes secretion of Dilp2 from brain insulin-producing cells (IPCs). Our data indicate that glial- and fat body-derived Dilp6 contribute to regulation of Dilp2. Right: Dilp2 suppresses output of Dilp6 from the larval fat body. **C)** Model of the roles of Dilp2 and Dilp6 in regulating nutrient storage and peripheral growth in late third instar larvae. Dilp2 secretion is stimulated by dietary protein. Dilp2 suppresses production of Dilp6, possibly via inhibition of Foxo. Dilp2 suppresses triglyceride storage on diets high in sucrose but promotes trehalose storage (63). Dilp6 secretion from fat body is regulated by ecdysone, insulin signaling, and dietary protein and sugar. Dilp6 secreted by the fat body promotes growth of peripheral organs like wing imaginal discs but limits triglyceride storage, possibly by directing the use of dietary sugar toward growth rather than lipogenesis. Both Dilp2 and Dilp6 promote whole-animal growth.
